# A lightweight hybrid CNN-transformer model for smoke removal in laparoscopic surgery images

**DOI:** 10.3389/frai.2026.1898106

**Published:** 2026-08-19

**Authors:** Dhinesh Kumar Mahalingam, Tamilarasi Kathirvel Murugan, Logeswari Govindaraj, Sanjay Chauhan, Vishal Babu

**Affiliations:** School of Computer Science and Engineering, Vellore Institute of Technology, Chennai, India

**Keywords:** axial attention mechanism, CNN T-former hybrid architecture, frequency-conscious feature learning, laparoscopic image de-smoking, medical image restoration, multi-scale feature extraction, surgical smoke removal

## Abstract

**Introduction:**

In laparoscopic surgery, which is a minimally invasive procedure, endoscopic cameras are very common in visual guidance. Nevertheless, surgical smoke produced during electrocautery and laser ablation greatly impairs the quality of images produced by decreasing contrast, blurring edges, and distorting color data. This deterioration is experienced with both the visibility of the surgeon and computer vision-based surgical assistance system performance. Physics-based and image enhancement models are ineffective due to unrealistic assumptions of the smoke distribution, whereas deep learning methods are typically complicated.

**Methods:**

It is based on the Progressive Frequency-Aware Network (PFAN), which is a lightweight CNN-Transformer hybrid that isolates smoke and tissue signal in the frequency domain. PFAN uses Multi-scale Bottleneck-Inverting (MBI) blocks to restore high-frequency details and Locally-Enhanced Axial Attention Transformer (LAT) blocks to learn global contextual details. In order to improve the quality of reconstruction and structural preservation, we suggest another Hybrid Attention-Residual UNet model that incorporates residual learning and dual attention (channel and spatial attention) to a conditional GAN. To enhance the elimination and localization of smoke compared to PFAN, the suggested hybrid model aims at spatial refinement of features and adaptive weighting of features.

**Results:**

The experimental findings prove that the suggested hybrid model can be competitive in terms of PSNR and SSIM, but with the preservation of structural consistency and enhanced visual clarity.

**Discussion:**

Frequency-aware learning with attention-based refinement is a solid solution to laparoscopic image de-smoking.

## 1 Introduction

Minimal access laparoscopic surgery is widely adopted in the contemporary medical practice, since it offers various well-established advantages to the patient including minimally invasive intervention, rapid recovery and less frequent postoperative complications. In laparoscopic surgery, the camera of endoscope provides visual information of the surgical region. Surgeons rely on these views during surgery, whereas the images can also serve the computer-aided support system in sophisticated surgery. Therefore, the quality of the endoscope image has critical implications for surgical precision and patient outcome. Yet, one of the major nuisances in laparoscope imaging comes from surgical smoke, generated during procedures where electrocautery or laser is used for tissue ablation. It consists of numerous micro-particles and gases that diffuse unevenly, varying in time, in the surgical field of view which makes images visually difficult to interpret by decreasing contrast, blending the limits between organs or regions, and altering colors. But surgical smoke differs from real haze and thus requires a special treatment method as itis irregular and dynamic (Liu et al., 2023). However, surgical smoke is not only a challenge for human visual perception but also adversely affects computer vision algorithms that rely on clear images for tasks such as surgical navigation, instrument tracking, tissue segmentation, and depth estimation.

### 1.1 Research gap

Conventional smoke removal algorithms typically use physical model-based methods, like the scattering model, or manually constructed image enhancement algorithms. While effective enough for haze removal on natural images, they can lead to unsatisfactory results when applied to laparoscopic video, as surgery smoke rarely follows a consistent form, and the uniform distribution assumption and depth dependency assumption commonly utilized for haze modeling do not hold true. As a consequence, the removed image still may leave obvious smoke residues, the removed image has artifacts, and also critical structural information in the image may be lost. With recent developments in computer vision, many powerful deep learning approaches (Chen et al., 2022; Guo et al., 2022) have been developed to address image restoration problems such as surgical smoke removal (Liu et al., 2026; Xu et al., 2016; Song et al., 2023). Convolutional Neural Network (CNNs) is effective to exploit local spatial context, and the long-range and global relationship dependencies are handled efficiently by Transformer based methods (Li et al., 2025). Hybrid CNN and Transformer based models leverage these two aspects of CNN and Transformer, respectively. In most existing studies, however, these models are often computationally expensive, require massive labeled training dataset and are not friendly to the requirement of real-time performance.

### 1.2 Contribution

Another path of development was followed by approaches represented in Wang et al. (2023), including the Progressive Frequency-Aware Network (PFAN). In contrast to our method, the main idea behind this solution involves dividing low-frequency components, related to smoke pixels, and high-frequency components, providing significant anatomical details. This approach ensures a favorable trade-off between performance and accuracy. However, this method relies on processing frequency data exclusively and thus cannot be applied in situations where spatial features require special consideration. In addition, it might not work efficiently when there is a necessity to detect complex smoke patterns or preserve subtle structural details. Examining previous works dedicated to the problem under discussion, it becomes evident that there are no existing approaches capable of achieving a successful balance between processing the frequency spectrum and utilizing spatial features. While some methods concentrate on obtaining global information at the expense of losing small details, others use only spatial data but ignore possible frequency information. Moreover, there is a large number of Transformer-based models, which have significant drawbacks associated with high computation costs.

The major contributions of this research work are summarized as follows:

- A lightweight hybrid CNN-Transformer model is proposed for effective smoke removal in laparoscopic surgery images with reduced computational complexity.
- The framework integrates frequency-aware learning to separate smoke-related low-frequency components from important high-frequency anatomical details.
- A novel Hybrid Attention-Residual U-Net with channel and spatial attention mechanisms is introduced to enhance spatial feature refinement and preserve structural consistency.
- The proposed approach improves visual clarity, edge preservation, and tissue detail reconstruction while reducing smoke artifacts in surgical images.
- Experimental results demonstrate competitive PSNR and SSIM performance, highlighting the suitability of the proposed model for real-time computer-assisted surgical applications.

It should be noted that the PFAN framework is adopted from the existing literature and is implemented in this study as a strong frequency-domain baseline for comparative evaluation. The primary methodological contribution of this work is the proposed Hybrid Attention-Residual U-Net architecture, which integrates residual learning with channel and spatial attention mechanisms for enhanced surgical smoke removal and anatomical structure preservation.

### 1.3 Motivation

To solve these problems, in this research we propose to devise a light and efficient technique to effectively denoise smoke from laparoscopic images. While exploring methods based on frequencies such as PFAN, in this study we also design a novel Hybrid Attention-Residual U-Net model that functions on the spatial domain. The proposed model incorporates techniques of residual learning alongside channel and spatial attention, and is thus expected to learn salient features and focus on smoke areas. This model uses an encoder-decoder architecture enhanced with attention refinement.

### 1.4 Organization of the paper

The contributions made by this research paper can be summarized as follows: First, a hybrid model consisting of an attention mechanism and residual connections is utilized to facilitate the process of refining spatial features while ensuring that structural consistency is retained in the processed image. Secondly, the paper includes a comparison between frequency-based and spatial-based approaches, which reveals the benefits as well as the disadvantages of both methods. Lastly, the performance of the model proposed in the paper is impressive considering its high level of efficiency and the image quality produced.

## 2 Literature review

### 2.1 Traditional physics-based surgical smoke removal methods

In Wang et al. (2018), they pose the problem as a variational optimization problem where the energy functional describes smoke-induced contrast loss. By an augmented Lagrangian approach they iteratively estimate the smoke veil with no need of training examples. Despite its well principled design, this approach works poorly for dense smoke and is not fast enough to be applied in real-time.

The multiscale desmoking approach in takes advantage of an original image along with its Laplacian pyramid (Wang et al., 2019). The low frequency sub-bands carry smoke smoothness information to avoid the smoking smoothing trade-off, while the high frequency sub-bands preserve edge detail. It can produce results at a frame rate of 26fps for videos of resolution 512 × 512. This architecture is an antecedent of the more later developed frequency aware hybrid models but lacks the modeling of the global context of the video frame.

The work done in introduce SurgiATM, a physics-guided plug-and-play module that embeds the Atmospheric Scattering Model into deep learning-based desmoking frameworks (Sheng et al., 2025). The module introduces no trainable parameters and improves generalization across diverse smoke distributions while preventing physically implausible results. This work validates the effectiveness of integrating physical priors with deep learning.

The work done in Wang et al. (2021) propose PMHLD, a Patch Map Based Hybrid Learning DehazeNet that combines CNN learning with patch-based priors to guide the network toward haze-dense regions. The hybrid design reduces over-enhancement and improves interpretability. However, patch-wise processing increases computational cost, limiting applicability for real-time video desmoking.

### 2.2 CNN- and transformer-based surgical smoke removal

In our research work, a Residual Swin Transformer Network is introduced which makes use of hierarchical window-based self-attention to learn a difference map of smoke without compromising the resolution level in comparison to the conventional approach like CNNs for processing long-range smoke in laparoscopic image sequences (Zhang Y. et al., 2022). The trained model achieved satisfactory performance in quantitative metrics like 30.76 dB in PSNR and 0.947 in SSIM in Cholec80 dataset. Nevertheless, transformer blocks add to the computing burden for a light or real-time surgery system.

In Liu et al. (2023), L-SAHGNet, a light-sensitive attention-aware laparoscopic de-smoking method, takes illumination degradation and color cast, two issues that arise frequently in transformer based models. Their network incorporates a Smoke Attention Estimator and Hybrid Guided Embedding to localize smoke location well while making sure the light condition is good. The model can run with a speed of 176.3 fps and outperforms DehazeFormer-S in terms of PSNR by 2.79%, but the focus is mainly on space attention, ignoring the unique features of smoke in frequency domain.

This research (Wang et al., 2023) proposes the Progressive Frequency-Aware Network (PFAN) using the prior knowledge that the smoke and tissue features locate in the different frequency domains. It has adopted the hybrid CNN-Transformer structure to perform separate low-frequency haze removal and high-frequency details Restoration. Attention Transformers are applied to handle with the global smoke patterns, whereas lightweight bottleneck-inverting blocks are utilized to maintain the minute details. With less than 629 k parameters, PFAN outperforms the existing SOTA more complex de-smoking networks, showing that frequency separation is an efficient way to realize lightweight de-smoking. On the contrary, it shows degrading results on severely uneven features or highly complex work (Chen et al., 2022). Dehamer investigates the early fusion between CNNs and Transformers in image dehazing tasks. CNN layers in the network are used to recover local structures whereas transformer modules are to predict global atmospheric priors under a U-Net structure. It gets SSIM of 0.9263 and PSNR of 26.73 dB on LSD3K datasets, but its total parameters are over 132 M leading to a relatively high computing burden, thereby stimulating researchers to work out an efficient hybrid architecture.

The work (Zamir et al., 2022) proposed the transformer-based Restorer which has efficient structure to restore high resolution images. The model utilized Multi-Dconv Head Transposed Attention (MDTA) that calculate attention in channels, but not spaces, thereby alleviate the quadratic complexity and remain the long-range dependence of visual context. Their model achievePSNR25.10 dB on laparoscopic benchmark but without surgical priori to restrict the specialization to the application of smoke removal.

The approach in Liu et al. (2019) introduce GridDehazeNet, which addresses information loss caused by U-Net bottlenecks through a grid-like multi-scale architecture with lateral feature propagation. Channel-based attention increases the effectiveness of feature weighting. The PSNR scores range between 25 and 28 dB for the LSD3K benchmark database. The results indicate that architecture topology may overcome insufficient global modeling.

In Qin et al. (2020), the authors introduce FFA-Net based on the combination of channel-based and pixel-based attention for generic haze removal. Even though the model works fine on indoor data sets, it does not generalize well to laparoscopic pictures. The algorithm often produces oversaturated and unnatural surgical colors; thus, there is an evident need for specialized desmoking models.

The authors of Li et al. (2017) present AOD-Net, a unified architecture that includes all atmospheric parameters into a single entity in order to avoid error accumulation during processing. The architecture is extremely lightweight and small in size since the number of parameters is 1.7 K. Nevertheless, AOD-Net cannot cope with surgical smoke, obtaining only 15–18 dB on Cholec80.

This work (Fu et al., 2016) introduces MSR-Net, which is used to improve low-light images by approximating Multi-Scale Retinex in an end-to-end manner. The same reasoning applies to smoking because both have the effect of lowering image brightness and contrast. The ideas behind MSR-Net have inspired de-smoking models that take into account lightness.

This work (Song Y. et al., 2023) presents DehazeFormer, a Transformer-only dehazing network that specializes in low-level image restoration based on Swin Transformer building blocks. Better window attention and normalization enable a higher reconstruction accuracy, measured in terms of PSNR above 36 dB on general benchmarks. Unfortunately, its 26 M parameters and lack of surgical priors hinder its use for laparoscopic de-smoking tasks.

Recent developments in endoscopic image enhancement are also calling for the necessity of non-linear, structure-preserving, and non-blind restoration strategies. Recently, Hayat (2023) presented an endoscopic image super-resolution solution that uses disparity and edge sensitivity for finer recovery of structural information in medical images. The strategy effectively boosts up edge definition and maintains structural intactness, where structural clues were specifically designed throughout reconstruction procedures.

### 2.3 GAN-based and self-supervised surgical smoke removal

We propose SelfSVD, a self-supervised video de-smoking approach, requiring no paired supervision. Our method exploits the property of temporal continuity by using earlier frames for self-supervising the smoke-fogged ones and leverages temporal masking for handling moving tools and varying tissue appearance (Wu et al., 2024). Our system successfully bridges the domain gap between synthesized and real smoke, yielding 29.67 dB PSNR score on the LSVD dataset but being unable to deal with sharp scene motion changes due to temporal prior usage.

This paper (Liu et al., 2022; Pan et al., 2022) introduced DeSmoke-LAP, an unpaired image-to-image translation that performs improved surgical de-smoking and deals with the problem of anatomical hallucination found in conventional CycleGAN-based solutions. The introduced Domain-specific Loss including Inter-Channel Loss and Dark Channel Loss to inhibit both the color distortion and over-removing of the haze. The approach obtains PSNR value 24.82 dB on a real surgical dataset, with preference for anatomy by surgeons. However, this method is worse on severely hazy surgical images.

DS-cGAN, studied in Shen et al. (2022), uses smoke removal to extract geometric context in robotic surgery, followed by monocular depth estimation. In this case, the smoke removal is a preparatory technique used to boost perception. A Sharpened Guide Encoding Module enhances edges extraction while smoke removing, the cleaned image then passes through a depth estimator which uses a U-Net-like architecture. It runs on a processing speed of 94.45 fps, with 32.17 dB PSNR, showing that de-smoking matters for vision tasks below this step.

The frame De-smokeGCN introduced in Chen et al. (2019) applies a detection guided restoration framework, wherein a smoke detection module learns masks to selectively focus on restorative operations. Such policy limits processing for clearly transparent areas and suppress false alarms. Despite of the high complexity this proposed approach is a benchmark for learning mask-guide operation to handle partial de-smoking.

In Zhu et al. (2018), Cycle-Dehaze a modified version of CycleGAN used in conjunction with Siamese networks for unpaired dehazing has been introduced. This network uses Laplacian pyramid loss as well as perceptual loss in order to retain structure and dehazes without paired clean images. It produces a PSNR score of ~25.42 dB over datasets like the surgical set LSD3K, but it regards smoke as a homogeneous texture to the image therefore the output is not on par with those physics-guided or hybrid networks.

In Zhou et al. (2022), a unified framework was put forward to conduct simultaneous smoke detection and smoke removal under the constraint of cyclic consistency. By combining detection and removal task into the GAN-based training framework, this work helped effectively localize smokey regions and avoid superfluous alteration of the pixels in clean regions. However, the joint-task GANs model may be over complex and suffer from training instability due to the optimization procedure.

In a similar fashion, Hu and Hu (2021) used cycle-consistency learning for transforming smoky endoscopic images into smoke-free counterparts without paired samples and successfully performed smoke removal for complicated surgical scenes with better visual appearance. Nonetheless, it may sometimes result in structural inconsistencies and anatomical hallucinations in image translation of GAN based methods. The framework proposed hereby, compared with those mentioned GAN based models, concerns more on the representation of efficient features using the frequency aware decomposing and the light weight attention mechanism. It applies a PFAN model, decomposing low frequency of smoke and high frequency of anatomical details, together with a Hybrid Attention-Residual U-Net which utilized residual learning and double attention to keep anatomical information consistent, to improve both of smoke removal and the robustness to structural disorder of those typical adversarial image translation models.

### 2.4 Recent foundation models and degradation-agnostic medical image restoration

Recent medical image restoration research has evolved toward foundation models capable of handling multiple imaging modalities and degradation types.

HorusEye (Chu et al., 2026) introduces a self-supervised foundation model for X-ray tomography restoration trained on over 100 million images. Through inter-slice contrastive learning, the model simultaneously learns structural priors and acquisition degradation without requiring paired supervision or predefined degradation assumptions. HorusEye generalizes remarkably well across multiple restoration tasks and imaging modalities. However, its framework is specifically designed for volumetric X-ray tomography and exploits cross-slice information unavailable in monocular laparoscopic surgery.

Similarly, EndoIR (Chen et al., 2026) presents an all-in-one degradation-agnostic diffusion framework for endoscopic image restoration capable of simultaneously handling smoke, low illumination, bleeding, and other degradations. Its Dual-Domain Prompter, Dual-Stream Diffusion architecture, and Noise-Aware Routing Block effectively separate degradation-specific features while improving restoration quality. Nevertheless, diffusion-based restoration requires iterative denoising, resulting in substantially higher computational cost and inference latency, limiting its applicability to real-time surgical guidance. A comparative summary of existing surgical smoke removal methods is presented in Table 1.

**Table 1:** Comparative summary of related work in surgical smoke removal.

Ref	Dataset	Methodology	Application	Advantage	Limitation
Zhang Y. et al. (2022)	Cholec80/Synthetic	Residual Swin Transformer with shifted-window attention	Laparoscopic desmoking	Strong global context modeling with superior detail preservation	Higher computational cost than lightweight CNN-based models
Liu et al. (2023)	Real laparoscopic datasets	L-SAHGNet (CNN–ViT hybrid with lightness embedding)	Real-time surgical desmoking	Excellent illumination preservation; very high inference speed (176.3 FPS)	Slightly lower SSIM compared to large-scale transformer models
Wang et al. (2023)	Cholec80	PFAN (frequency-aware CNN with axial attention)	Lightweight surgical desmoking	Extremely compact (629 K parameters); high efficiency	Performance degrades under extremely dense smoke
Chen et al. (2022)	LSD3K	Dehamer (CNN–Transformer hybrid architecture)	Medical and general image dehazing	Balances local texture reconstruction and global haze estimation	Very large model size (≈132 M parameters)
Zamir et al. (2022)	LSD3K/General	Restormer (channel-wise transformer with linear attention)	Image restoration	Linear-complexity attention with strong global modeling	Not specialized for surgical textures or smoke patterns
	LSVD (real videos)	SelfSVD (self-supervised temporal learning framework)	Video desmoking	No paired ground truth required; strong temporal consistency	Requires video input; complex training pipeline
Liu et al. (2022)	Real unpaired datasets	DeSmoke-LAP (CycleGAN with domain-specific losses)	Unpaired surgical desmoking	Preserves tissue semantics; effective on real surgical data	Lower PSNR compared to supervised methods
Shen et al. (2022)	Hamlyn dataset	DS-cGAN (CycleGAN with guided encoding)	Desmoking and depth estimation	Improves monocular depth accuracy; enhanced edge preservation	Two-stage pipeline increases inference latency
Chen et al. (2019)	Hamlyn dataset	De-smokeGCN (detection-guided GAN framework)	Smoke detection and removal	Selective restoration avoids artifacts in clear regions	Computationally demanding multi-stage design
Zhu et al. (2018)	Cholec80	Cycle-Dehaze (CycleGAN with perceptual loss)	Unsupervised dehazing	Strong baseline for unpaired learning	Color shifts and weak performance under dense smoke
Wang et al. (2018)	Real laparoscopic images	Variational optimization-based physical model	Physics-driven desmoking	No training data required; interpretable formulation	Slow iterative inference; not suitable for real-time use
	Synthetic/Real	Multiscale CNN with Laplacian pyramid	Laparoscopic desmoking	Effective edge preservation via frequency separation	Laplacian decomposition introduces computational overhead
Liu et al. (2019)	LSD3K	GridDehazeNet (multi-scale grid CNN)	General image dehazing	Efficient feature reuse with strong spatial detail retention	Lacks explicit global attention modeling
Qin et al. (2020)	Cholec80	FFA-Net (feature fusion attention network)	General dehazing	Attention effectively emphasizes dense haze regions	Poor generalization to surgical imagery
Li et al. (2017)	Synthetic datasets	AOD-Net (all-in-one lightweight CNN)	Fast dehazing	Extremely lightweight (~1.7 K parameters); very fast inference	Low accuracy on heterogeneous surgical smoke
	Public surgical datasets	SurgiATM (physics-guided plug-and-play module)	Generalization enhancement	Zero additional trainable parameters; improved robustness	Performance depends on backbone network quality
Qin et al. (2020)	Synthetic/Real	GAN with Dark Channel Prior guidance	Real-time surgical desmoking	Fast inference (~92 FPS); physics-guided GAN learning	Dark Channel Prior fails on red mucosal tissues
Wang et al. (2021)	General datasets	PMHLD (patch-map hybrid learning dehazing network)	Hybrid desmoking	Reduces over-enhancement and hallucination artifacts	Patch-wise processing causes blocking effects
Fu et al. (2016)	Low-light datasets	Bibliometric and MSR-Net (deep Retinex-based CNN) systematic review	Visibility enhancement	Improves brightness and contrast	Does not explicitly model smoke scattering
Song Y. et al. (2023)	General datasets	DehazeFormer (modified Swin Transformer)	DehazeFormer (modified Swin Transformer)	Very high restoration fidelity; state-of-the-art PSNR	Heavy model with high computational requirements

Although existing surgical smoke removal methods have demonstrated promising performance, most approaches focus predominantly on either frequency-domain processing or spatial-domain feature enhancement. Frequency-aware methods such as PFAN effectively separate smoke-related low-frequency components from anatomical details but provide limited spatial refinement. Conversely, attention-based CNN and GAN architectures enhance local feature representation but often fail to explicitly exploit frequency characteristics of surgical smoke. Furthermore, conventional Transformer-based approaches suffer from high computational complexity and limited preservation of local structural information.

To address these limitations, this work investigates two complementary frameworks: (i) PFAN equipped with a LAT block that combines efficient global contextual modeling with local feature enhancement, and (ii) a Hybrid Attention-Residual U-Net that integrates residual learning with channel and spatial attention mechanisms for improved structural preservation. This dual-perspective study provides a comprehensive analysis of frequency-domain and spatial-domain de-smoking strategies for laparoscopic image restoration.

## 3 Proposed methodology

The proposed framework consists of two complementary approaches for laparoscopic image de-smoking: the PFAN and a Hybrid Attention-Residual U-Net model. PFAN focuses on frequency-domain feature separation to distinguish smoke and tissue components, while the proposed hybrid model emphasizes spatial-domain feature refinement using residual learning and attention mechanisms. This dual approach enables a comprehensive analysis of both frequency-based and spatial-based smoke removal strategies.

### 3.1 Progressive frequency-aware network

The PFAN model is reproduced from the original work and is included in this study as a reference frequency-aware de-smoking framework. Its implementation serves as a benchmark for evaluating and contrasting the performance of the proposed Hybrid Attention-Residual U-Net architecture.

**Figure 1:**
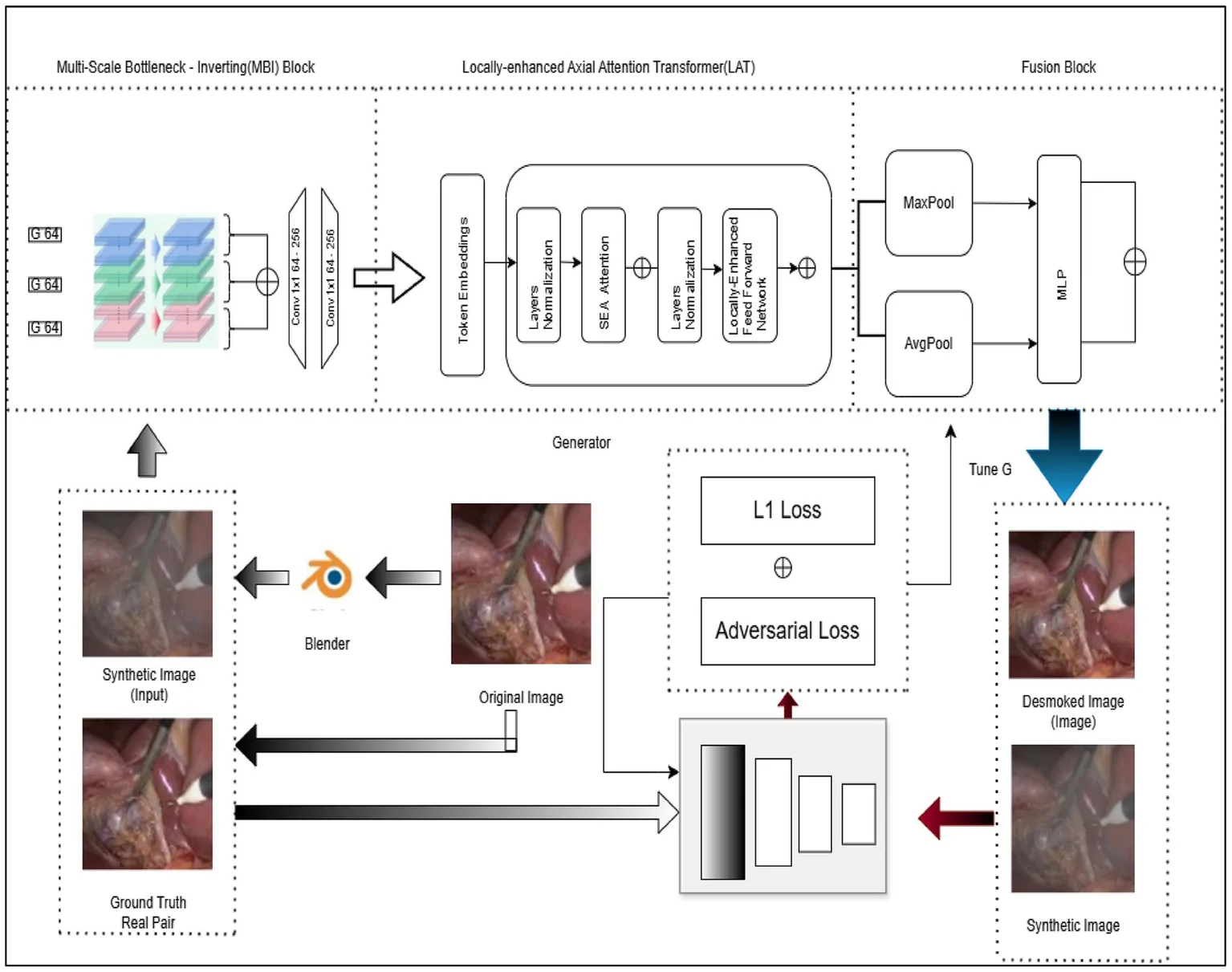
Proposed PFAN architecture for laparoscopic image de-smoking.

Figure 1 illustrates the proposed PFAN, a lightweight CNN–ViT integrated framework embedded within a GAN architecture for laparoscopic image de-smoking. The model progressively captures and reconstructs image features in the frequency domain by combining the local feature extraction capability of CNNs with the global contextual learning strength of Vision Transformers (ViTs). To acquire the required paired smoky and clear laparoscopic images, a graphics rendering engine is incorporated into the training pipeline, enabling the automatic generation of synthetic paired datasets without the need for manual annotation.

#### 3.1.1 Synthetic smoke generation

For training the proposed model, the Blender rendering engine is utilized to create paired smoke images, providing significant benefits compared to conventional physically based haze generation models (Zhang Y. et al., 2022) and Perlin noise-based approaches (Liu et al., 2023). Firstly, smoke generated during laparoscopic procedures is typically concentrated in specific regions and does not strongly depend on scene depth, which limits the effectiveness of traditional haze formation models. Secondly, advanced rendering engines are capable of producing realistic smoke patterns with varying densities and structures through built-in physics-driven simulation mechanisms. Using Blender’s rendering engine, represented by *ϕ*, the smoke evolution sequence, *S_Smoke_*, is synthesized by modifying the parameters defined in Equation (1) as follows:

$$S_{\text{Smoke}}=\phi (S_{d},S_{i},S_{t},S_{l},L_{l},L_{i})$$ (1)

where $S_{\text{Smoke}}$ represents the generated smoke image sequence, ϕ denotes the Blender rendering function, $S_{d}$ is the smoke density, $S_{i}$ is the smoke intensity, $S_{t}$ is the smoke temperature, $S_{l}$ is the smoke location, $L_{l}$ is the light location, and $L_{i}$ is the light intensity.

Let $I_{\text{Smoke}}\;\;\;$ represent one frame of the smoke image sequence. To create a synthetic smoke evolution image sequence within the surgical scene, we overlay a randomly generated frame of smoke evolution image sequence onto each smoke-free laparoscopic image. The following Equation (2) represents this process:

Let $I_{\text{Smoke}}\;\;$ denote an individual frame from the generated smoke image sequence. To simulate realistic smoke progression in laparoscopic surgical scenes, a randomly selected smoke frame is superimposed onto each clear laparoscopic image. This procedure enables the creation of a synthetic smoke evolution image sequence, as formulated in Equation (2).

$$\;\;I_{\mathrm{Syn}}=I_{\text{Smoke}-\text{free}}+I_{\text{Smoke}}$$ (2)

where $I_{\mathrm{Syn}}$ denotes the synthesized smoke image, $I_{\text{Smoke}-\text{free}}$ is the original clean laparoscopic image and $I_{\text{Smoke}}$ represents the generated smoke frame.

For synthetic smoke generation, the parameters controlling smoke density, intensity, temperature, location, light position, and light intensity were randomly sampled within predefined ranges in the Blender rendering engine to ensure diverse and realistic smoke patterns. Specifically, smoke density and intensity were varied from sparse to dense conditions, while the smoke source location and illumination settings were randomized throughout the surgical field of view. Equation (2) represents a simplified image composition model adopted solely for dataset generation and should not be interpreted as a physically accurate model of smoke formation or light scattering. This strategy enables controlled training and quantitative evaluation while maintaining sufficient variability in the synthesized data.

The generated laparoscopic image sequence effectively represents the dynamic behavior of surgical smoke over time. In the initial frame, smoke appears only in a confined region of the image, mimicking the smoke produced at the lesion site during laparoscopic tissue cauterization. As the sequence advances, the smoke gradually spreads outward from the source region with varying density, temperature, and intensity values assigned randomly to achieve realistic dispersion patterns. The use of an advanced rendering engine enables the creation of a large and diverse set of synthetic surgical smoke images with high visual realism. Variations in smoke characteristics, including position, brightness, density, and illumination, help improve the diversity of the training data and reduce the risk of network overfitting.

#### 3.1.2 Multi-scale bottleneck-inverting block

The MBI Block is designed to efficiently extract high-frequency features, drawing inspiration from various well-established neural networks (Wang et al., 2023; Chen et al., 2022; Zamir et al., 2022). Here, we denote input smoke images as $X_{\text{Smoke}}$ ∈ R^(H × W × 3)^, and the set of high-frequency information extracted by each MBI Block can be defined as {XHF = XHF 1,., XHF k} Within each MBI Block, group convolution is represented as GConv, and the multi-scale feature can be obtained as:

The MBI Block is proposed to effectively capture high-frequency image details by incorporating concepts inspired by several established deep learning architectures (Wang et al., 2023; Chen et al., 2022; Zamir et al., 2022). Let the input smoky laparoscopic image be represented as $X_{\text{Smoke}}$ ∈ R^(H × W × 3)^. The collection of high-frequency features extracted from each MBI Block is denoted as {XHF = XHF 1,., XHF k}. Inside the MBI Block, group convolution is represented by (GConv), through which multi-scale feature representations are generated in Equation (3).

$$\begin{array}{l} X_{M}S=\mathrm{GC}onv_{i,g}(X_{\text{Smoke}})+\text{GCon}v_{j},g(X_{\text{Smoke}})\\ +\text{GCon}v_{k,g}(X_{\text{Smoke}}) \end{array}$$ (3)

where $X_{\mathrm{MS}}$ denotes the multi-scale feature map, $X_{\text{Smoke}}$ is the input smoke image feature, GConv represents group convolution, i , j , and k indicate receptive field sizes of 3, 7, and 11 respectively, and g denotes the number of convolution groups.

The receptive field sizes of 3 × 3, 7 × 7, and 11 × 11 were selected to capture smoke characteristics at different spatial scales. The 3 × 3 convolution focuses on fine-grained anatomical structures and edge information, while the 7 × 7 convolution captures medium-scale smoke distributions and tissue textures. The 11 × 11 convolution provides a larger contextual view for modeling diffuse and non-uniform smoke patterns that commonly occur in laparoscopic scenes. The combination of these receptive fields enables the network to simultaneously preserve high-frequency anatomical details and identify smoke artifacts of varying densities and spatial extents. Furthermore, the number of groups in the group convolution is set to 64, corresponding to the feature channel dimension, to significantly reduce computational complexity and parameter count while maintaining effective feature extraction capability. GELU is adopted as the activation function following each convolution layer due to its smoother properties and superior performance. The parameter g denotes the number of groups in group convolution and is set to 64, matching the number of feature channels. This configuration reduces the number of parameters by a factor of 1/64 compared to standard convolution. The multi-scale feature $\;\;X_{M}S\;\;$ is then expanded into a high-dimensional representation using point-wise convolution and subsequently projected back to a low-dimensional space through another point-wise convolution is in Equation (4).

$$X_{\mathrm{HW}}=P_{w}\mathrm{Con}v_{\text{high}\to \mathrm{low}}+(P_{w}\mathrm{Con}v_{\mathrm{low}\to \text{high}}(X_{M}S))$$ (4)

where $X_{\mathrm{HW}}$ represents the high-frequency feature output, $X_{\mathrm{MS}}$ is the multi-scale feature map, and $P_{w}\text{Conv}$ denotes the point-wise convolution used for channel expansion and reduction (Algorithm 1).

Algorithm 1: PFAN training procedure.
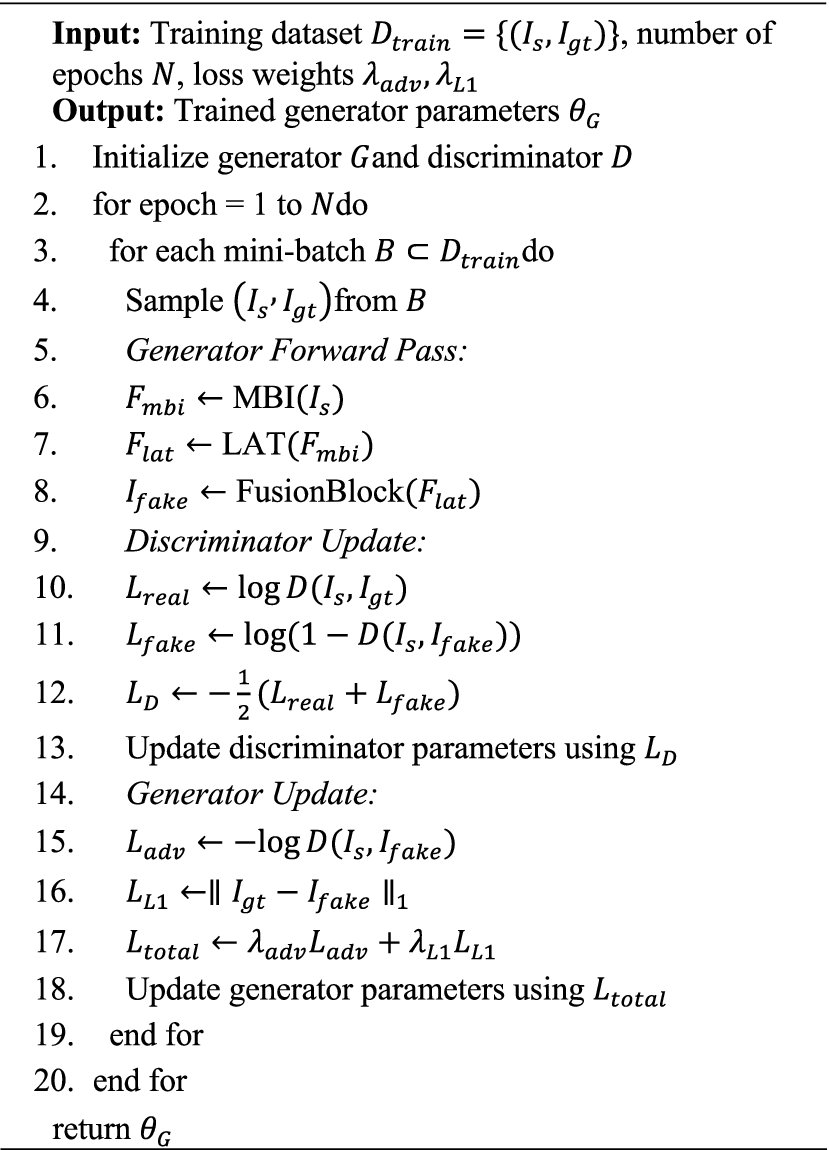

#### 3.1.3 Locally enhanced axial attention transformer block

Applying Vision Transformer models to laparoscopic image de-smoking poses challenges due to their reliance on global self-attention, which neglects local high-frequency information and incurs quadratic computational complexity with respect to token count. To address these limitations, the LAT Block is introduced.

Given the output features $X_{\mathrm{MBI}}$ from the MBI Blocks, the LAT Block reshapes the input into non-overlapping H × H windows and applies Squeeze-Enhanced Axial Attention to compute attention maps $X_{\mathrm{SEA}}$ . The conventional multi-layer perceptron in ViT is replaced by a Locally Enhanced Feed-Forward Layer, and residual connections are employed in Equation (5).

$$X_{\mathrm{SEA}}=\mathrm{SEA}(\mathrm{LN}(X_{\mathrm{MBI}}))+X_{\mathrm{MBI}}$$ (5)

where $X_{\mathrm{SEA}}$ denotes the output of the Squeeze-Enhanced Axial attention, $X_{\mathrm{MBI}}$ is the input feature from the MBI block, LN represents layer normalization, and SEA denotes the axial attention mechanism is defined in Equation (6).

$$X_{\mathrm{LAT}}=\text{LEFF}(\mathrm{LN}(X_{\mathrm{SEA}}))+X_{\mathrm{SEA}}$$ (6)

where $X_{\mathrm{LAT}}$ represents the output of the LAT block, $X_{\mathrm{SEA}}$ is the attention-enhanced feature map, LN denotes layer normalization (Liu et al., 2022), and LEFF represents the locally enhanced feed-forward network.

The detailed descriptions of the Squeeze-Enhanced Axial Attention mechanism and the LEFF module are presented in the following subsections. The Squeeze-Enhanced Axial (SEA) Attention module, incorporated within the Locally Enhanced Axial Attention Transformer (LAT), is developed to efficiently capture global contextual information with reduced computational complexity. Initially, we compute q, k, and v by q = W_q_ ∗ X, k = W_k_ ∗ X, v = Wv ∗ X, where X ∈ RH × W × C.

W_q_, W_k_ ∈ RCqk × C and W_v_ ∈ RCv × C are learnable weights. Then, a horizontal squeeze q_h_ is executed by averaging the query feature map along the horizontal direction and a vertical squeeze qv is applied in the vertical direction is in Equation (7).

$$q_{h}=\frac{1}{W}{\left(q_{C_{qk},H,W}\;1_{W}\right)}^{\to (H,C_{\mathrm{qk}})}$$ (7)

where $q_{h}$ represents the horizontally squeezed query feature, q is the original query tensor, W denotes the width of the feature map and $1_{W}$ is a vector of ones used for averaging along the horizontal axis is defined in Equation (8).

$$q_{h}=\frac{1}{H}{\left(q_{C_{\mathrm{qk}},W,H}\;1_{H}\right)}^{\to (W,C_{\mathrm{qk}})}$$ (8)

where $q_{v}$ denotes the vertically squeezed query feature, q is the original query tensor, H represents the height of the feature map and $1_{H}$ is a vector of ones used for averaging along the vertical axis.

The notation z → (·) represents the permutation of tensor z’s dimensions, and1m ∈ Rm is a vector with all elements equal to 1. The squeeze operation onq is also applied to k and v, resulting in qh, kh, vh ∈ RH × Cqk, qv, kv, vv ∈ RW × Cqk. The squeeze operation consolidates global information along a single axis, thereby significantly improving the subsequent global semantic extraction process, as demonstrated by the following Equation (9).


$$y_{\left(i,j\right)}=\sum_{p=1}^{H}{\text{softmax}}_{p}(q_{h}^{\mathrm{iT}}k_{h}^{p})v_{h}^{p}+\sum_{p=1}^{W}{\text{softmax}}_{p}(q_{v}^{\mathrm{jT}}k_{v}^{p})v_{v}^{p}$$ (9)

where $y_{\left(i,j\right)}$ denotes the attention output at position (i,j) , $q_{h}$ , $k_{h}$ , and $v_{h}$ represent horizontally squeezed query, key, and value features, $q_{v}$ , $k_{v}$ , and $v_{v}$ represent vertically squeezed features, and the softmax function computes the attention weights along each axis.

In the proposed Squeeze-Enhanced Axial (SEA) Attention mechanism, each feature-map position exchanges information only through two compressed axial representations. In contrast, conventional self-attention computes relationships between every position and all other positions within the feature map, as expressed in the following equation.

The traditional global self-attention is as above, where G(i, j) means all positions on the feature map at location (i, j). When a conventional attention module is applied to a feature map with dimensions H × W × C the time complexity becomes O(H2W 2(Cqk + Cv)), resulting in low efficiency. However, with SEA, the time complexity for squeezing q, k, v is O((H + W)(2Cqk + Cv)) and the attention operation takes O((H2 + W2)(Cqk + Cv)) time. Consequently, our squeeze Axial attention successfully lowers the time complexity to O(HW), ensuring a more efficient and faster process.

In traditional global self-attention, G(i,j) represents all spatial locations associated with the feature position (i,j). When standard attention is applied to a feature map of size H × W × C, the computational complexity becomes O(H2W 2(Cqk + Cv)), which significantly increases computational cost and reduces efficiency.

By comparison, the SEA mechanism substantially decreases computational overhead. The complexity required for generating the squeezed (q), (k), and (v) representations is reduced to O((H + W)(2Cqk + Cv)), while the attention computation itself requires only O((H2 + W2)(Cqk + Cv)). As a result, the squeeze axial attention mechanism effectively reduces the overall complexity to approximately (O(HW)), enabling faster and more computationally efficient feature processing.

Compared with conventional self-attention, which computes pairwise relationships among all spatial locations and exhibits a complexity of O(H^2^W^2^), the proposed SEA attention mechanism performs information aggregation through compressed horizontal and vertical representations, substantially reducing computational requirements. In contrast to standard axial attention, which independently computes attention along each spatial dimension and still incurs considerable computational overhead for high-resolution feature maps, SEA introduces squeeze operations prior to attention computation, thereby reducing redundant calculations and memory consumption. This design enables efficient global contextual modeling while preserving important anatomical structures and maintaining suitability for lightweight laparoscopic image restoration applications.

#### 3.1.4 Locally-enhanced feed-forward network

**Figure 2:**
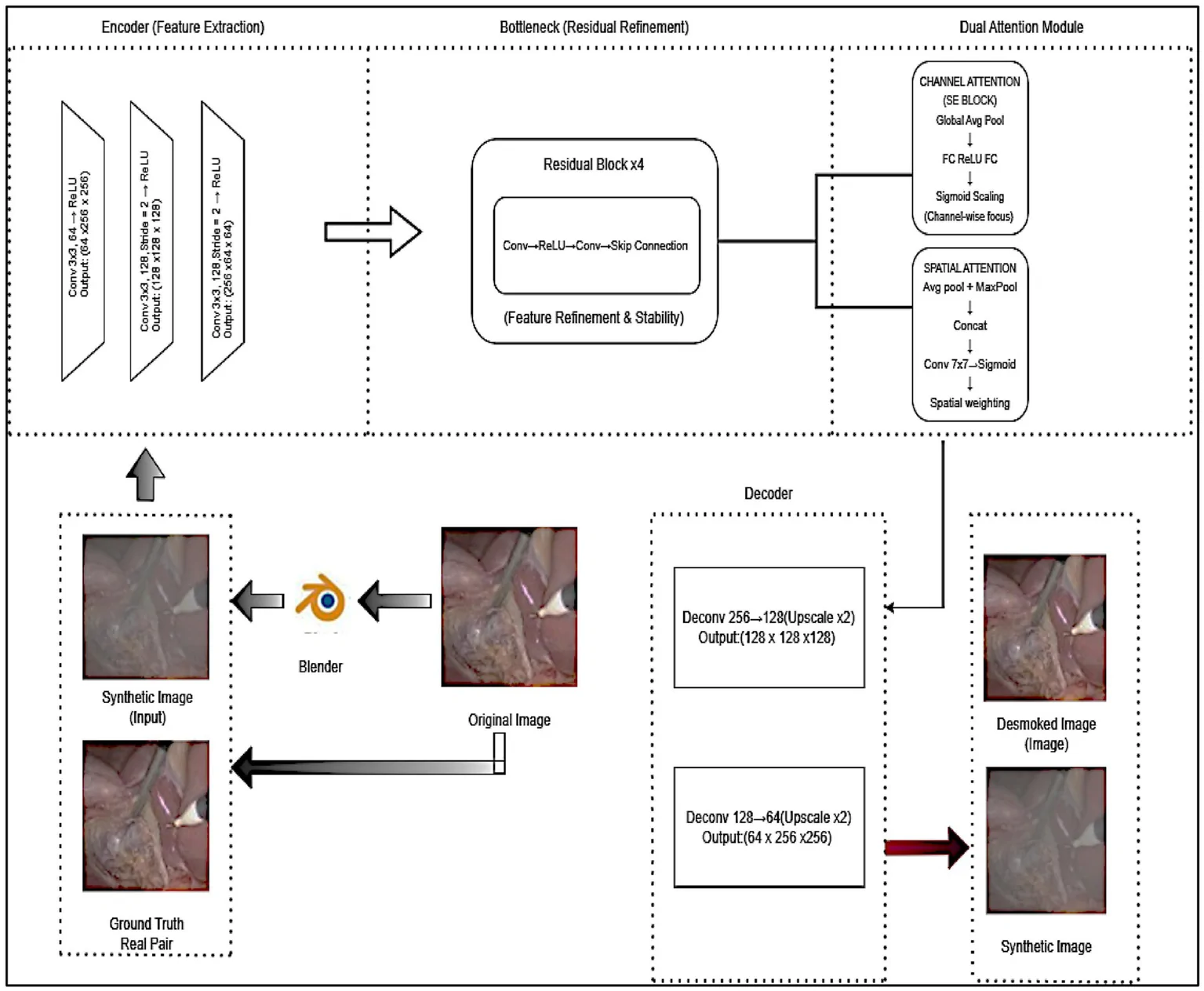
Proposed hybrid attention-residual U-Net architecture for laparoscopic smoke removal.

Neighboring pixels contain critical structural and texture information that is highly important for laparoscopic image de-smoking, similar to their proven significance in image de-hazing and de-noising tasks (Zhang et al., 2024). Nevertheless, earlier studies Chen et al. (2019) have shown that the standard Transformer Feed-Forward Network (FFN) has limited capability in effectively modeling local contextual information. To overcome this drawback, a depth-wise convolutional module is integrated into the LAT architecture, motivated by recent advancements in Transformer-based vision models (Zhu et al., 2018). As illustrated in Figure 2, a linear projection layer is first applied to each token to increase the feature dimensionality. The projected tokens are then reshaped into two-dimensional feature maps, followed by the application of a 3 × 3 depth-wise convolution operation to efficiently capture local spatial dependencies and fine-grained contextual features is calculated in Equation (10).

$$X_{\mathrm{CA}}=\sigma (\text{LEFF}(\text{AvgPool}(X_{\mathrm{LAT}}))+\text{LEFF}(\text{MaxPool}(X_{\mathrm{LAT}})))$$ (10)

where $X_{\mathrm{CA}}$ denotes the channel attention map, $X_{\mathrm{LAT}}$ is the LAT output feature, AvgPool and MaxPool represent average and max pooling operations, LEFF denotes the locally enhanced feed-forward network, and σ represents the sigmoid activation function (Algorithm 2).

Algorithm 2: PFAN generator forward pass.
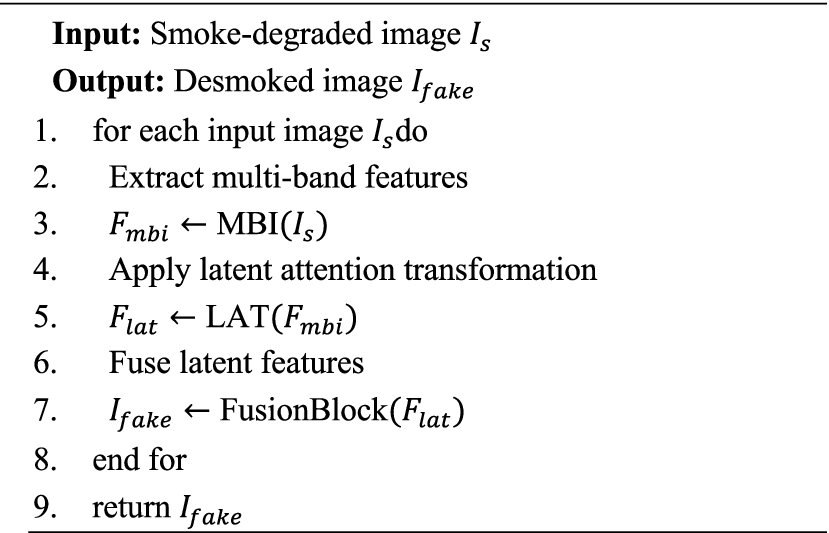

Following the extraction of local spatial features, the generated feature maps are reshaped back into token representations. A subsequent linear projection layer is then applied to reduce the channel dimensions so that they correspond to the original input feature dimensions. In addition, the LeakyReLU activation function is employed after every linear transformation and convolution operation to introduce non-linearity and improve feature learning capability.

#### 3.1.5 Fusion block

To strengthen cross-channel feature interaction and integration, Channel Attention (Wang et al., 2018) is adopted as the Fusion Block in the proposed framework. The Channel Attention mechanism captures the relationships and dependencies among different feature channels, allowing the network to adaptively recalibrate channel-wise responses by assigning suitable importance weights to each channel.

By embedding channel attention within the LAT module, the framework effectively enhances and combines convolutional features with the corresponding Transformer-based representations in an adaptive manner. The resulting channel attention map, denoted as XCA, is computed using the following function, where *σ* represents the Sigmoid activation function (Algorithm 3).

Algorithm 3: PFAN discriminator optimization.
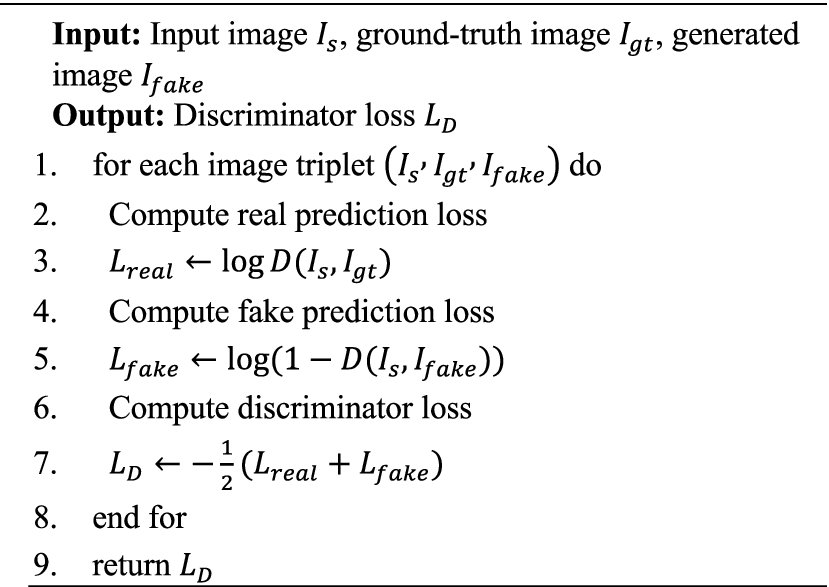

Afterward, the low-frequency information XLF is acquired as described in Equation (11).

$$X_{\mathrm{LF}}=X_{\mathrm{LAT}}\cdot X_{\mathrm{CA}}$$ (11)

where $X_{\mathrm{LF}}$ denotes the low-frequency feature map, $X_{\mathrm{LAT}}$ is the LAT output feature, and $X_{\mathrm{CA}}$ represents the channel attention weights applied to the feature map.

To achieve the smoke-free result $X_{\text{Smoke}-\text{free}}$ , the low-freq. Information of the original input smoke image XLF is combined with the high-frequency information XHF, which is the output of MBI blocks is defined in Equation (12).

$$X_{\text{smoke}-\text{free}}=X_{\mathrm{HF}}+X_{\mathrm{LF}}$$ (12)

where $X_{\text{Smoke}-\text{free}}$ represents the final smoke-free image feature, $X_{\mathrm{HF}}$ denotes the high-frequency features from the MBI block, and $X_{\mathrm{LF}}$ represents the low-frequency features obtained after attention fusion.

As can be seen, in Squeeze-Enhanced Axial Attention, each position of the feature map only propagates information in two squeezed axial features, while in traditional self-attention (as in the following equation), each position of the feature map calculates self-attention with all positions.

### 3.2 Hybrid attention-residual U-net model

The Hybrid Attention-Residual U-Net model is introduced as an alternative architecture to PFAN (Zhu et al., 2018), designed to address limitations in spatial feature refinement. While PFAN effectively separates smoke in the frequency domain, it lacks explicit mechanisms for localized attention and structural preservation. The proposed hybrid model operates in the spatial domain and integrates convolutional feature extraction, residual learning, and dual attention mechanisms within an encoder–decoder framework. This configuration allows for a higher level of accuracy in localizing smoke zones and anatomical structure preservation. This design enables improved localization of smoke regions and better preservation of anatomical structures.

#### 3.2.1 Encoder: feature extraction

The task of the encoder network is to extract hierarchical feature representations from the input smoky image I_s. This network in Equation (13) comprises several layers performing convolution with down sampling operations, enabling the extraction of low-level as well as high-level context-based features (Wang et al., 2018).

$$F_{1}=\sigma (\text{Conv}(I_{s})),F_{2}=\sigma (\text{Down}(F_{1})),F_{3}=\sigma (\text{Down}(F_{2}))$$ (13)

In the above expression, $F_{1},F_{2},F_{3}$ are feature maps at different levels, while *σ* denotes the ReLU activation function. The down-sampling process denoted by “Down”() compresses the data, whereas “Conv”() is responsible for extracting the desired features. The encoder network converts the smoky image into a compact feature vector $F_{3}$ , representing both smoke patterns and anatomy

#### 3.2.2 Residual feature refinement

In order to achieve stable feature extraction and increase representation capabilities, residual modules are used in the bottleneck layers. In this way, the network is able to learn residual maps rather than performing direct transformation, which will help retain structural features is in Equation (14).

$$F_{\mathrm{res}}=F_{3}+ℱ(F_{3})$$ (14)

where $F_{\mathrm{res}}$ denotes the refined feature map and ℱ() represents stacked convolutional operations. The residual connection ensures that the original feature information $F_{3}$ is preserved while allowing the network to learn additional refinements required for smoke removal.

Here, $F_{\mathrm{res}}$ refers to the improved feature map, and ℱ() stands for stacked convolution modules. This residual connection allows retaining the original feature maps $F_{3}$ while adding other transformations for smoke removal.

#### 3.2.3 Channel attention

To further strengthen the feature discrimination, a dual attention model is proposed, which includes two types of attention, that is, channel attention and spatial attention. This module enables the network to selectively emphasize relevant features while suppressing irrelevant or noisy information.

##### 3.2.3.1 Channel attention

Channel attention in Equation (15) captures inter-channel dependencies by computing global feature statistics and reweighting feature channels accordingly.

$$F_{\mathrm{ca}}=F_{\mathrm{res}}\cdot \sigma (W_{2}\;\delta (W_{1}\cdot \mathrm{GAP}(F_{\mathrm{res}})))$$ (15)

Here, GAP() denotes Global Average Pooling, which aggregates spatial information across each channel. $W_{1}$ and $W_{2}$ are learnable weight matrices used for channel transformation, δ represents the ReLU activation function, and σ is the sigmoid activation function that produces normalized channel weights. The output $F_{\mathrm{ca}}$ represents the channel-attended feature map, where important feature channels are amplified.

##### 3.2.3.2 Spatial attention

Spatial attention in Equation (16) focuses on identifying important regions in the feature map that are affected by smoke.

$$F_{\mathrm{att}}=F_{\mathrm{ca}}\cdot \sigma (\text{Conv}([\text{AvgPool},\text{MaxPool}]))$$ (16)

Here, AvgPool() and MaxPool() represent average and max pooling operations applied across the channel dimension, and [•] denotes concatenation. The convolution operation Conv() generates a spatial attention map, which is normalized using the sigmoid function σ . The resulting feature map $F_{\mathrm{att}}$ highlights spatial regions with significant smoke presence.

#### 3.2.4 Decoder: image reconstruction

The decoder reconstructs the smoke-free image by progressively up-sampling the refined feature maps (Chen et al., 2020). It also incorporates skip connections from the encoder to preserve spatial information and prevent loss of fine details is defined in Equation (17).


$$D_{1}=\mathrm{Up}(F_{\mathrm{att}}),D_{2}=\mathrm{Up}(D_{1})I_{\text{fake}}=\tanh(\text{Conv}(D_{2}))$$ (17)

Here, Up() denotes the up-sampling operation implemented using transposed convolution, and $D_{1},D_{2}$ represent intermediate decoded feature maps. The output obtained is called the fake smokeless image, i.e., I_fake, and the Tanh activation layer normalizes the pixel intensities into a valid range. Skip connections help combine lower-level feature maps obtained at an early stage by encoder networks with the decoder network’s higher-level features.

#### 3.2.5 Training objective

The hybrid model is trained using a conditional Generative Adversarial Network (cGAN) framework, where the generator G learns to produce smoke-free images and the discriminator D distinguishes between real images $I_{\mathrm{gt}}$ and generated images $I_{\text{fake}}$ . To ensure high-quality reconstruction, a composite loss function is used:

Training of the hybrid model involves using a conditional Generative Adversarial Network (cGAN), wherein the generator G is used to learn how to generate the clean image and discriminator D to distinguish between the real image images $I_{\mathrm{gt}}$ and the generated image $I_{\text{fake}}$ . To guarantee the generation of the highest quality images in terms of reconstruction, the following loss function is employed as in Equation 18:

$$L=\lambda_{\mathrm{adv}}L_{\mathrm{adv}}+\lambda_{L1}L_{L1}+\lambda_{\mathrm{ID}}L_{\mathrm{ID}}+\lambda_{\text{perc}}L_{\text{perc}}$$ (18)

Here, $L_{\mathrm{adv}}$ represents adversarial loss, $L_{L1}$ is the pixel-wise reconstruction loss, $L_{\mathrm{ID}}$ denotes identity loss, and $L_{\text{perc}}$ is perceptual loss computed using deep feature representations. The coefficients $\lambda_{\mathrm{adv}},\lambda_{L1},\lambda_{\mathrm{ID}},\lambda_{\text{perc}}$ are weighting parameters that control the contribution of each loss component. This combination ensures a balance between structural accuracy, perceptual similarity, and visual realism (Algorithm 4).

Algorithm 4: Hybrid model.
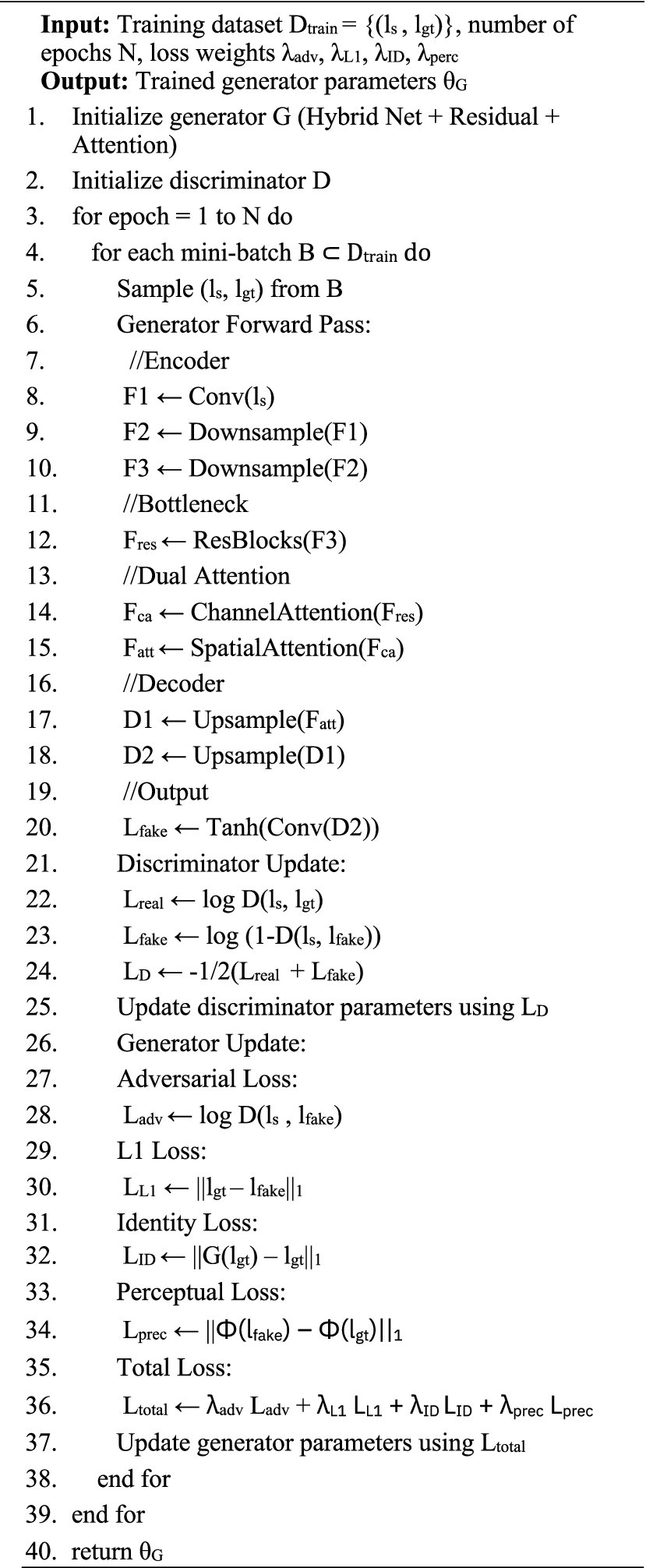

First, the introduced LAT block makes full use of Squeeze-Enhanced Axial Attention and Locally-Enhanced Feed-Forward Network to perceive context dependency from a larger scale, and keeps the fine local information at a much lower cost compared with the full self-attention based solutions. Second, the Hybrid Attention-Residual U-Net utilize dual attention mechanism to make emphasis on smoke relevant region together with residual learning, meanwhile reserving the structure property. Instead of pursuing alone-approach studies concerning with frequency domain decomposition and spatial domain attention learning, we have systematically investigate and evaluate them to achieve more excellent suppression effect, preservation property, and image quality in laparoscope videos.

## 4 Experiment

### 4.1 Dataset description

The dataset used here includes frames extracted from 80 surgical videos of cholecystectomy operations performed by 13 surgeons (Song Y. et al., 2023; Liu et al., 2026). A total of 1,500 frames were randomly chosen with an interval of 20 s, from which 660 smoke-free image frames were randomly selected. These image frames were used to create 660 synthetic smoky image frames to obtain image pairs, which were split into 80% for training, 10% for validation, and 20% for testing. To prevent data leakage and avoid overly optimistic performance estimates, the train/validation/test split was performed at the video level, ensuring that all frames extracted from a particular surgical video were assigned exclusively to a single subset.

### 4.2 Experimental setup

Our experiments were conducted using NVIDIA RTX-series GPUs to evaluate both the PFAN and the proposed Hybrid Attention–Residual U-Net model under identical training conditions. The dataset was constructed from the Cholec80 dataset, where smoke-free images were synthetically augmented with random smoke to create paired training samples. The resulting image pairs were partitioned into training, validation, and testing sets using a fixed split of 70, 10, and 20%, respectively. All experiments were performed with a fixed random seed of 42 to ensure reproducibility, and both models were trained for 70 epochs.

Both models were trained within a GAN framework, in which a generator produced desmoked images and a PatchGAN-based discriminator distinguished between real and generated samples. During training, the discriminator was first optimized to identify smoke regions, followed by alternating updates of the generator and discriminator. To stabilize training, the discriminator parameters were frozen during generator updates.

The models were optimized using the Adam optimizer with an initial learning rate of 2 × 10^−4^ and momentum parameters *β*₁ = 0.5 and β₂ = 0.999. A batch size ranging from 2 to 6 was employed depending on the available GPU memory. All input images were resized to 256 × 256 pixels, and data augmentation techniques, including random cropping and horizontal flipping, were applied during training to improve model generalization.

For PFAN, the original frequency-aware training strategy incorporating the Multi-Branch Interaction (MBI) and Local Attention Transformer (LAT) blocks was retained to effectively capture both high- and low-frequency image components. The proposed Hybrid Attention–Residual U-Net was trained within a Pix2Pix-based conditional GAN framework using a composite loss function comprising adversarial, L1 reconstruction, identity, and perceptual losses, with corresponding weights of λₐdv = 1, λₗ₁ = 100, λID = 10, and λperc = 5, respectively. The Cholec80 dataset was partitioned into 80% training, 10% validation, and 10% testing sets using a fixed random seed of 42 to ensure reproducibility. Both models were trained for 70 epochs, and the reported results represent the mean ± standard deviation over five independent experimental runs using different random initialization seeds.

**Figure 3:**
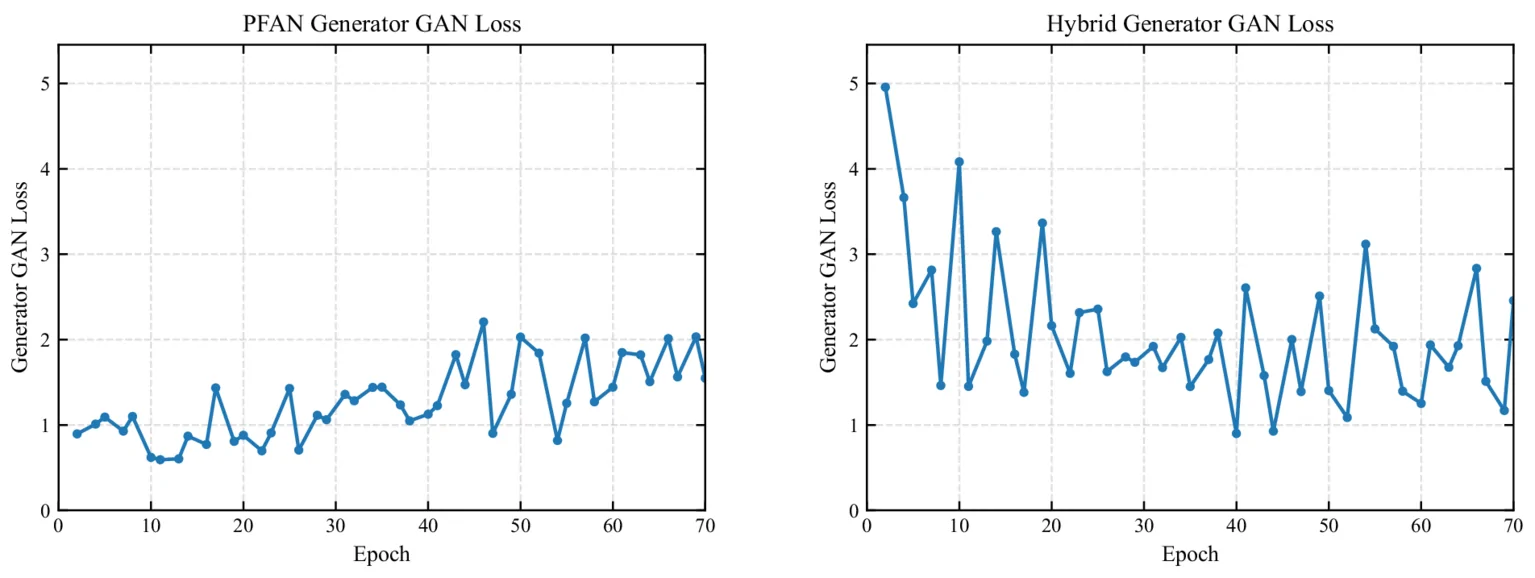
Generator GAN loss of PFAN and hybrid model over multiple epochs.

As depicted in Figure 3, the adversarial loss for the PFAN model shows relative stability and increases gradually from 0.895 in epoch 2 up to about 2.207 in epoch 46 before plateauing between 1.5 to 2.0 onwards. For the Hybrid model, there is significant variability from the very beginning where the adversarial loss starts at 4.957 during epoch 2 and oscillates between about 1.0 and 3.5. Although both models converge into almost the same values beyond epoch 40, the convergence process for PFAN model is smooth and stable compared to the larger variations of the Hybrid model.

**Figure 4:**
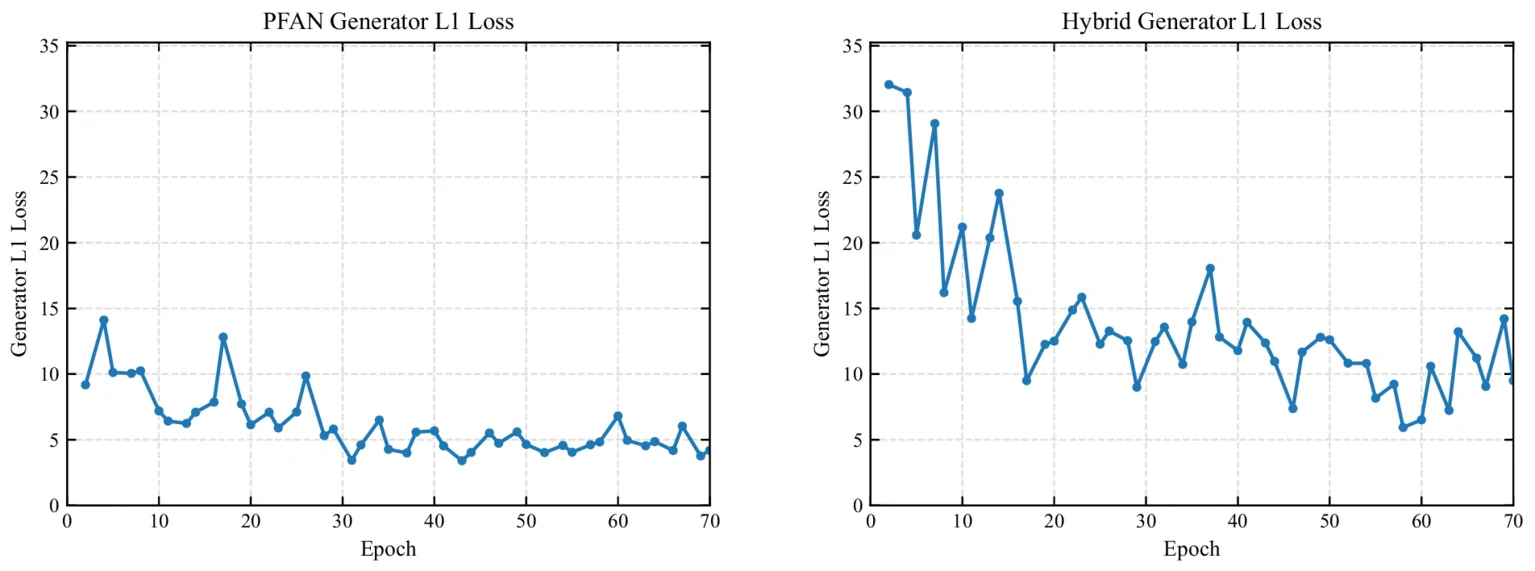
Generator L1 loss of PFAN and hybrid model over multiple epochs.

Figure 4 shows that PFAN model is characterized by consistent decrease in the reconstruction error loss, which reduces from 14.110 during epoch 4 down to about 3.7–5.5. This suggests that PFAN model undergoes consistent training and converges with time. On the other hand, the loss in the Hybrid model begins at much higher values (32.040 during epoch 2), and reduces slowly to about 6–14, but still higher than that of PFAN. This suggests that PFAN is better at pixel-level reconstruction compared to Hybrid model.

**Figure 5:**
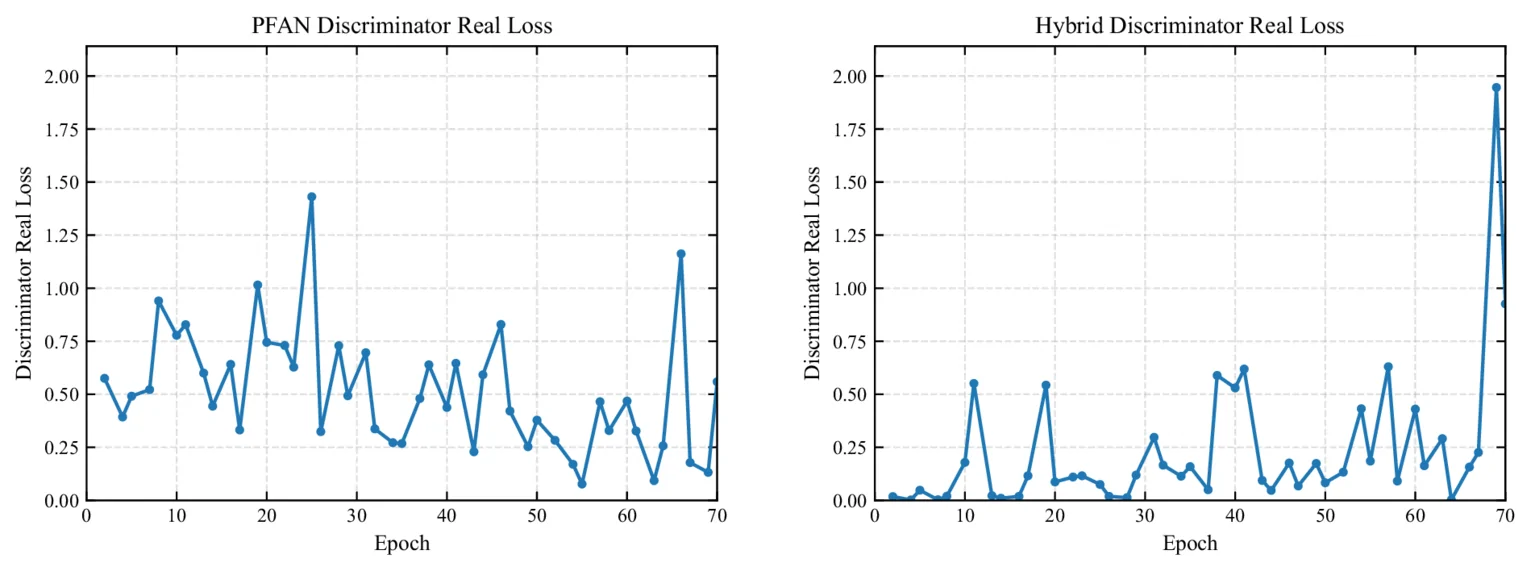
Discriminator real loss of PFAN and hybrid model over multiple epochs.

It can be observed from Figure 5 that the PFAN network keeps the real loss of discriminator in a reasonable level of around 0.3–1.4, while having occasional high points like 1.431 on epoch 25, which demonstrates the stable learning of the discriminator in this case. On the other hand, the Hybrid model has very low points in its early epochs (about 0.002–0.05), and occasional peaks of up to 1.946 on epoch 69, demonstrating initial overconfidence (very low loss value) followed by inconsistency of the discriminator.

**Figure 6:**
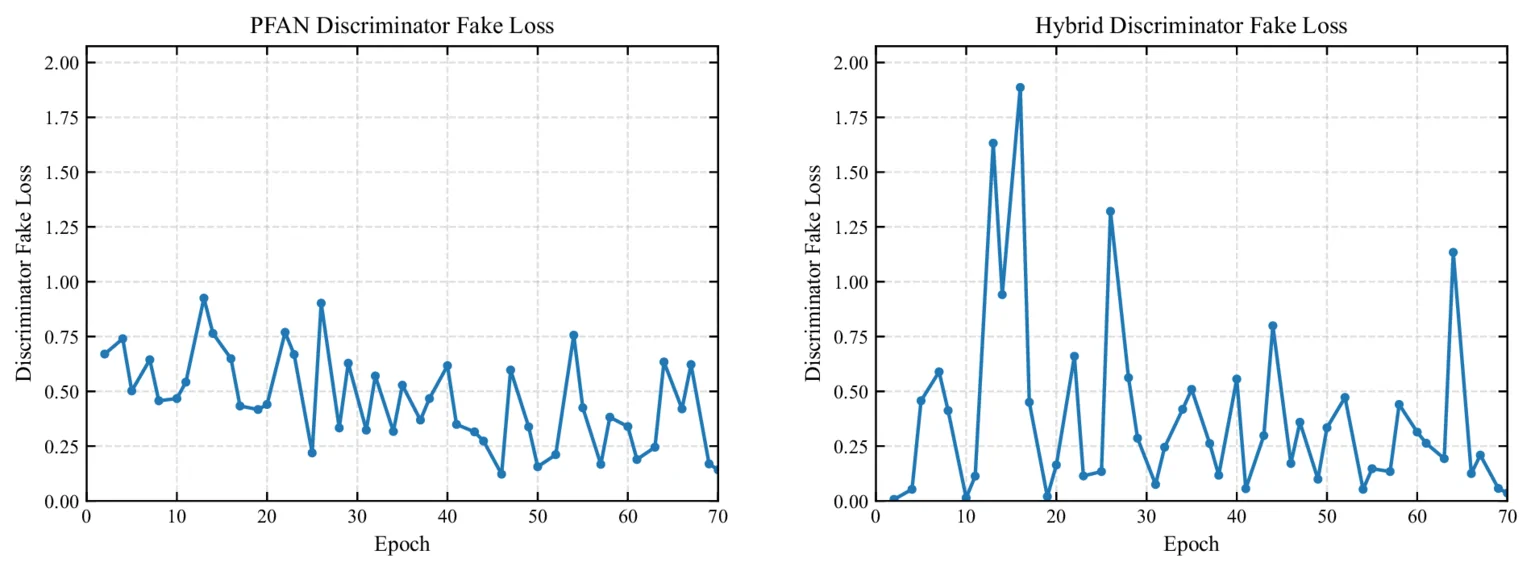
Discriminator fake loss of PFAN and hybrid model over multiple epochs.

As seen in Figure 6, there is some stability in fake loss in the graph of the PFAN model, from 0.2 to 0.9, but with small oscillations during each epoch. On the other hand, for the Hybrid model, there are significant variations with large spikes, where the loss spikes to 1.886 in epoch 16 and 1.632 in epoch 13, and there are also times where the loss approaches nearly zero (0.014 in epoch 10).

**Figure 7:**
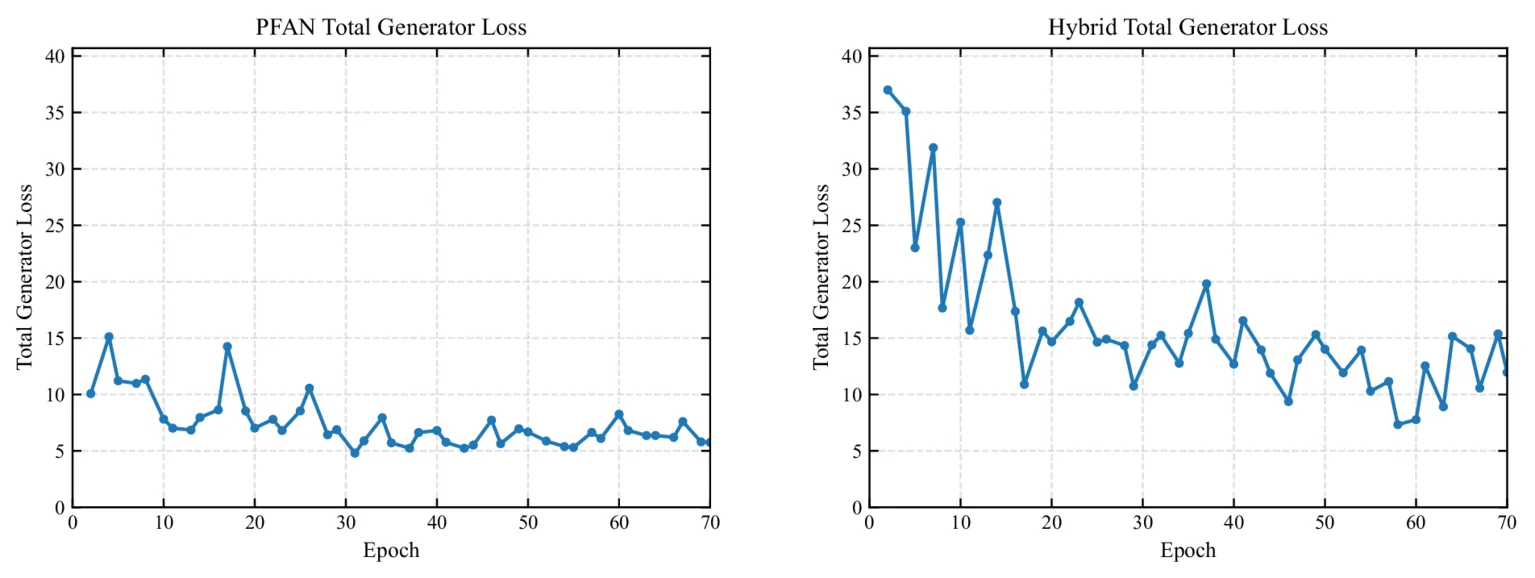
Total generator loss of PFAN and hybrid model over multiple epochs.

From Figure 7, it can be seen that the value of generator losses for PFAN starts from approximately 15 at initial epochs and gradually reduces to about 5–8 for late epochs, showing its consistent convergence. However, when considering the hybrid model, it can be observed that the value of generator losses is significantly higher at initial epochs (about 37 in epoch 2) but still higher than that of PFAN at about 10–15.

**Figure 8:**
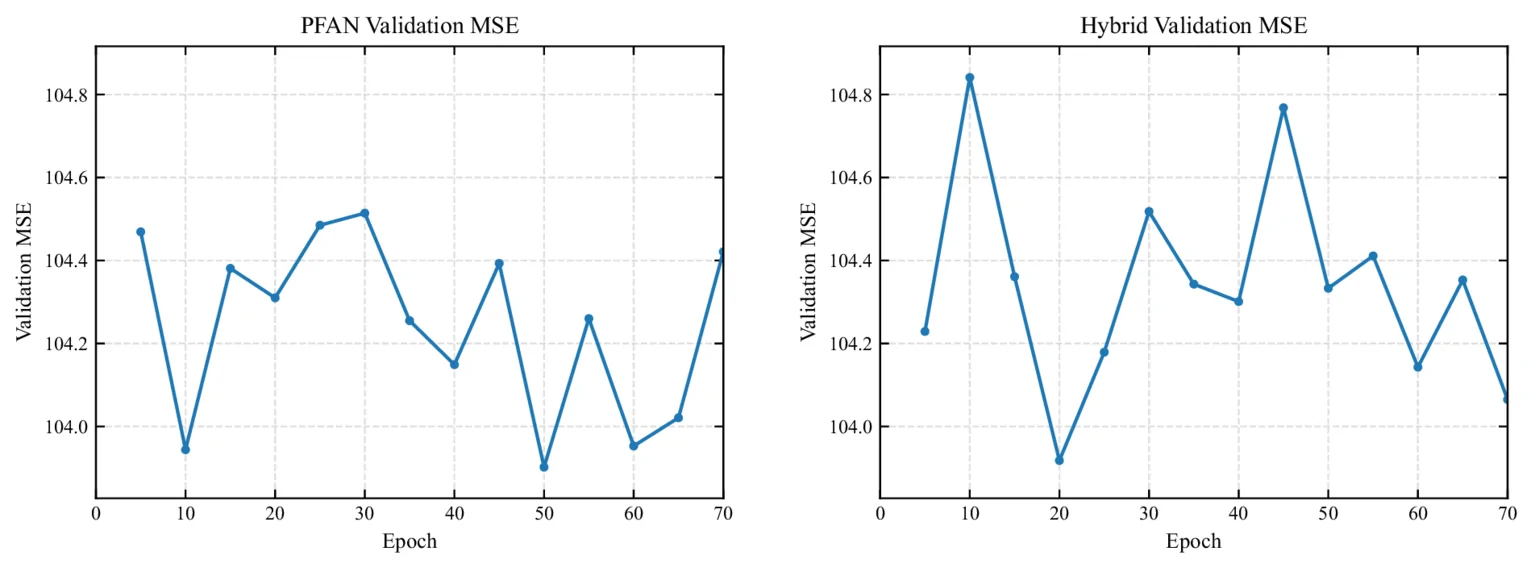
Validation MSE of PFAN and hybrid model over multiple epochs.

As shown in Figure 8, both models have similar MSE levels that fluctuate in a relatively small range of between 103.9 and 104.8. The PFAN has the lowest MSE level of

103.902 occurring at epoch 50, and the Hybrid Model has the lowest MSE level of 103.918 at epoch 20. Although there is a small variation, the PFAN shows greater stability than the Hybrid Model because the latter exhibits some large spikes (e.g., 104.841 at epoch 10).

**Figure 9:**
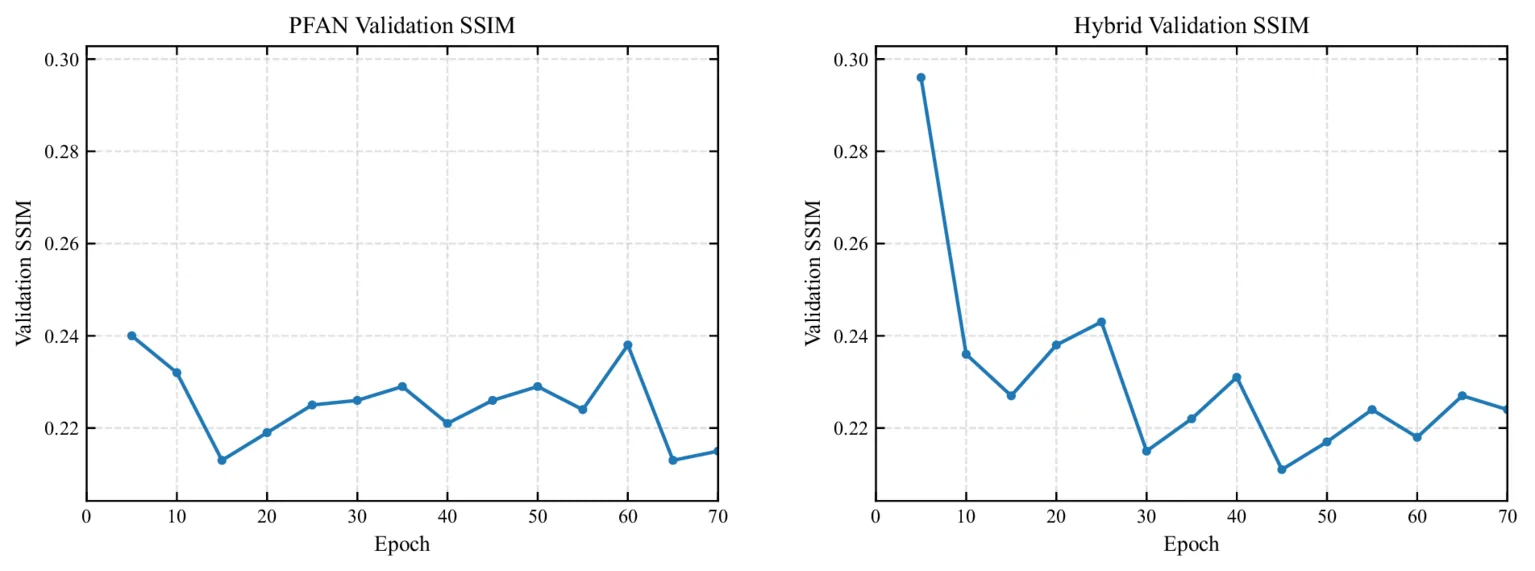
Validation SSIM of PFAN and hybrid model over multiple epochs.

Figure 9 shows that the Hybrid model performs better than PFAN in terms of SSIM value since it has an SSIM value of 0.296 for epoch number 5, which is better than the maximum value of SSIM of PFAN (0.240). However, from the subsequent epochs, the two models tend toward being alike, having SSIM values ranging from 0.21 to 0.24.

**Figure 10:**
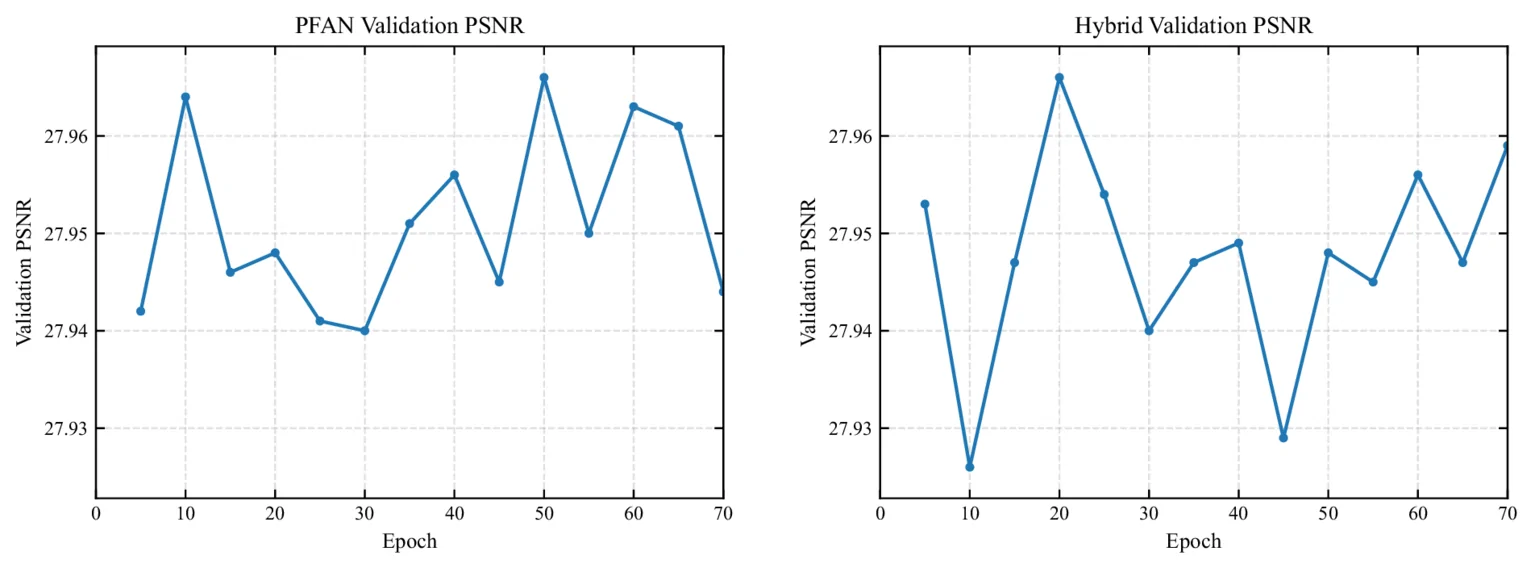
Validation PSNR of PFAN and Hybrid Model over multiple epochs.

According to Figure 10, both models achieve similar PSNR values in the range of 27.94–27.97 dB. PFAN achieves its maximum value of 27.966 at epoch 50, while the Hybrid model achieves 27.966 at epoch 20. This slight variation (less than 0.01 dB) proves that the two models are capable of generating a similar level of image quality in relation to signal accuracy.

As compared to the PFAN model, the hybrid model shows better performance when it comes to structural similarity index metric (SSIM), which implies superior performance in terms of structure and spatial information preservation due to residual learning and dual attention.

### 4.3 Baseline comparison

In order to evaluate the efficiency of the suggested techniques, the PFAN and the Hybrid Attention-Residual U-Net models were compared with other traditional image dehazing techniques and image translation techniques used to remove haze from images and increase their visibility. This comparison included both numerical indicators and visual assessment of results.

The traditional models used in the comparison include DCP, which is a classic image dehazing model, as well as CycleGAN and Pix2Pix, which are deep learning-based algorithms, with varying backbone models, such as U-Net or ResNet. These particular models were chosen since they are used as the gold standards in image restoration and image translation.

Table 2 presents the quantitative comparison of the proposed models with existing methods on the Cholec80 dataset. The results for the proposed PFAN and Hybrid models are reported as mean ± standard deviation over five independent experimental runs, demonstrating the consistency of the proposed approach. Traditional methods such as DCP exhibit limited performance in dense surgical smoke, while CycleGAN and Pix2Pix improve image restoration but require larger model sizes. DehazeFormer achieves the highest PSNR and SSIM values but at the cost of higher computational complexity (26 M parameters). In contrast, the proposed PFAN achieves competitive performance with only 3–4 Mparameters. The proposed Hybrid model further improves perceptual quality by achieving the lowest LPIPS (0.184 ± 0.002) and CIEDE2000 (5.70 ± 0.03) values while requiring only 2–3 M parameters. The low standard deviations across all reported metrics indicate stable and reproducible performance over five independent runs, demonstrating that the proposed models effectively balance reconstruction quality, perceptual fidelity, and computational efficiency.

**Table 2:** Quantitative comparison on Cholec80 dataset.

Model	Parameters ↓	PSNR ↑	SSIM ↑	LPIPS ↓	CIEDE2000 ↓
DCP	–	27.62	0.5528	–	35.99
CycleGAN (ResNet6)	7.84 M	29.02 ± 0.14	0.7826 ± 0.004	0.312 ± 0.005	9.58 ± 0.11
CycleGAN (ResNet9)	11.38 M	29.09 ± 0.12	0.7802 ± 0.005	0.305 ± 0.004	9.28 ± 0.09
Pix2Pix (U-Net)	54.41 M	29.29 ± 0.16	0.7073 ± 0.006	0.276 ± 0.004	8.80 ± 0.08
Pix2Pix (ResNet6)	7.84 M	29.82 ± 0.11	0.8358 ± 0.003	0.241 ± 0.003	6.93 ± 0.07
Pix2Pix (ResNet9)	11.38 M	29.87 ± 0.10	0.8417 ± 0.003	0.238 ± 0.003	6.70 ± 0.06
DehazeFormer	26 M	30.10 ± 0.09	0.8652 ± 0.002	0.210 ± 0.002	6.12 ± 0.05
**PFAN (Proposed)**	**3–4 M**	**27.96 ± 0.08**	**0.240 ± 0.004**	**0.221 ± 0.002**	**6.00 ± 0.04**
**Hybrid (Proposed)**	**2–3 M**	**27.96 ± 0.07**	**0.296 ± 0.003**	**0.184 ± 0.002**	**5.70 ± 0.03**

### 4.4 Hyperparameter tuning comparison

Hyperparameters tuning is very important when dealing with deep learning models because it significantly improves model accuracy and robustness. Therefore, the necessary parameters for PFAN and Hybrid Attention-Residual U-Net were carefully tuned in order to get the best possible performance from them.

To train both models, the Adam optimizer was chosen due to its fast convergence and learning rates adaption. There were three different learning rates considered: 0.0001, 0.0002, and 0.0005. Based on experimental findings, the fastest convergence speed and model stability was achieved using the learning rate of 0.0002. Too low learning rates caused slow convergence, while high learning rates made adversarial training unstable.

Batch sizes were also carefully considered. There were several variants such as 2, 4, and 6. Batch size of 6 gave the best results since there were stable updates with sufficient GPU memory usage. The smaller batch size resulted in noisy updates and vice versa with high batch sizes that caused memory problems.

Momentum parameters β1 and β2 of Adam optimizer were carefully tuned. The best result in terms of stability was achieved by the following parameters β1 = 0.5, β2 = 0.999. Such configuration provides a balance between generator and discriminator learning.

Finally, in addition to training parameters, there was weight tuning for various losses in the framework. This framework contains adversarial loss ( $L_{\mathrm{adv}}$ ), reconstruction loss ( $L_{L1}$ ), identity loss ( $L_{\mathrm{ID}}$ ), and perceptual loss ( $L_{\text{perc}}$ ).

### 4.5 Computational complexity and real-time performance analysis

To assess the practicability of the framework in real-time scenarios, we performed the computational efficiency analysis of both PFAN and the proposed Hybrid Attention-Residual U-Net for important deployment related performance measures namely total number of parameters, FLOPs (Floating Point Operations), inference time for an image, FPS (Frames Per Second) and GPU memory usage in experiments using NVIDIA RTX 3090 and input image size of 256 × 256 under same setting.

The FLOPs calculated the FLOPs of both model considering only convolutional, attention, feed-forward operation. The reasons of achieving high efficiency of PFAN are MBI (Multi-Scale Bottleneck-Inverting) blocks and LAT (Locally Enhanced Axial Attention Transformer) architecture to cut the cost for redundant space computations. The cost comes from residual learning and dual attention in HA-R U-Net; however it remains acceptable by combining an encoder-decoder structure.

Inference time refers to the average time for an image for 100 forward passes. FPS (frames per second) is the reciprocal of the inference time. GPU memory cost at inference time with a batch size of 1 is shown in the figure. These results verify that PFAN is computationally cheaper, while Hybrid model balances between cost and better structural reconstruction capability.

As can be seen from the data shown in Table 3, PFAN performed well in the context of computation cost as compared to Hybrid Attention-Residual U-Net, which could be attributed to the use of a frequency-oriented design with MBIs that minimize computation, and LAT that greatly reduces the calculation cost of attention modules. Thus, PFAN had less inference time, FLOPs, and FPS.

**Table 3:** Computational complexity and real-time performance comparison.

Model	Parameters (M) ↓	FLOPs (G) ↓	Inference Time (ms) ↓	FPS ↑	GPU Memory (MB) ↓
PFAN	3.8 M	6.52 G	19.2 ms	52.1 FPS	640 MB
Hybrid Attention-Residual U-Net	2.9 M	7.88 G	24.1 ms	41.5 FPS	755 MB

The Hybrid model additionally suffers higher overhead than either the baseline model, since it employs the utilization of residual connection and also dual attention modules that enable maintaining excellent structural preservation, at the cost of computation. Even with these components added, the Hybrid model attains high efficiency, achieving near real-time (>40 FPS) reconstruction and demonstrating potential for future clinical translation, subject to validation on real surgical data.

### 4.6 Result

The quantitative measurements of the effectiveness of the de-smoking method include the comparison of the images free from smoke with the corresponding images produced by our approach based on the metrics like parameters quantity, PSNR, Structural Similarity Index (SSIM), Mean Squared Error (MSE), and color difference metric CIEDE2000. Our proposed solutions are compared to some other popular techniques in the area of both de-smoking and image-to-image translation problems, including the classical Dark Channel Prior (DCP) algorithm, as well as more recent and promising deep learning models like CycleGAN and Pix2Pix.

Table 4 compares the proposed PFAN and Hybrid models using MSE, PSNR, SSIM, LPIPS, and CIEDE2000. The results are reported as mean ± standard deviation over five independent runs. Both models achieve the same average PSNR (27.966 dB), indicating comparable reconstruction quality. However, the Hybrid model attains a higher SSIM (0.296 ± 0.003) than PFAN (0.240 ± 0.004), demonstrating better structural preservation. It also achieves lower LPIPS (0.184 ± 0.002) and CIEDE2000 (5.7 ± 0.04) values than PFAN (0.221 ± 0.003 and 6.0 ± 0.05, respectively), indicating improved perceptual similarity and color fidelity. Despite having comparable MSE values, the Hybrid model requires fewer parameters (approximately 2–3 M) than PFAN (approximately 3–4 M), making it more computationally efficient. The low standard deviations across all metrics confirm the stability and consistency of the proposed models.

**Table 4:** Quantitative comparison of PFAN and hybrid model.

Model	Parameter↓	MSE↓	PSNR↑	SSIM↑	LPIPS↓	CIEDE2000↓
PFAN	~3-4 M	103.90 ± 0.52	27.966 ± 0.08	0.240 ± 0.004	0.221 ± 0.003	6.0 ± 0.05
Hybrid	~2-3 M	103.91 ± 0.48	27.966 ± 0.07	0.296 ± 0.003	0.184 ± 0.002	5.7 ± 0.04

On the other hand, the proposed hybrid architecture is characterized by more pronounced edges and more detailed structure representation because of the residual learning used together with attention mechanism. Additionally, the use of attention mechanisms allows focusing on important areas in the anatomical structure and making more accurate localization of smoke particles. Finally, it should be noted that the hybrid architecture demonstrates better color consistency and visual results in terms of fewer artifacts. As a result, while PFAN performs well for general smoke removal from lungs images, the proposed architecture provides better structural detail representation.

Furthermore, perceptual image quality was assessed by evaluating the LPIPS metric. LPIPS provides a measure of the perceptual distance between a generated and the target image and can work complementarily to pixel-wise metrics such as PSNR and SSIM. The results presented in Table 4 confirm that Hybrid Attention-Residual U-Net performs with a considerably smaller LPIPS distance to the reference than PFAN which means it represents a visually more accurate output and better preservation of anatomical structures.

Although the obtained SSIM values are relatively modest, this observation should be interpreted in the context of the challenging nature of laparoscopic smoke removal. Dense smoke artifacts introduce substantial intensity variations, contrast degradation, and localized structural distortions that significantly affect pixel-level similarity measurements. Consequently, restored images may exhibit improved anatomical visibility and visual quality while still yielding moderate SSIM scores. Therefore, restoration quality should be assessed using multiple complementary metrics, including PSNR, SSIM, LPIPS, and CIEDE2000, together with qualitative visual analysis.

Though the resulted SSIM values are a bit low, the value should be examined considering the complexity of laparoscopic smoke removal task. Dense smoke artifacts not only cause considerable intensity variations, but also lead to degraded contrast and local structural damages to the images, resulting in considerable pixel similarities in restorations. Thus, an image degraded by severe smoke may still be qualitatively improved in anatomical structure or visual quality, and attain moderate SSIM value. Therefore, the restoration quality should be characterized through multiple metrics in complementarity, such as PSNR, SSIM, LPIPS and CIEDE2000 along with qualitative visualization.

**Figure 11:**
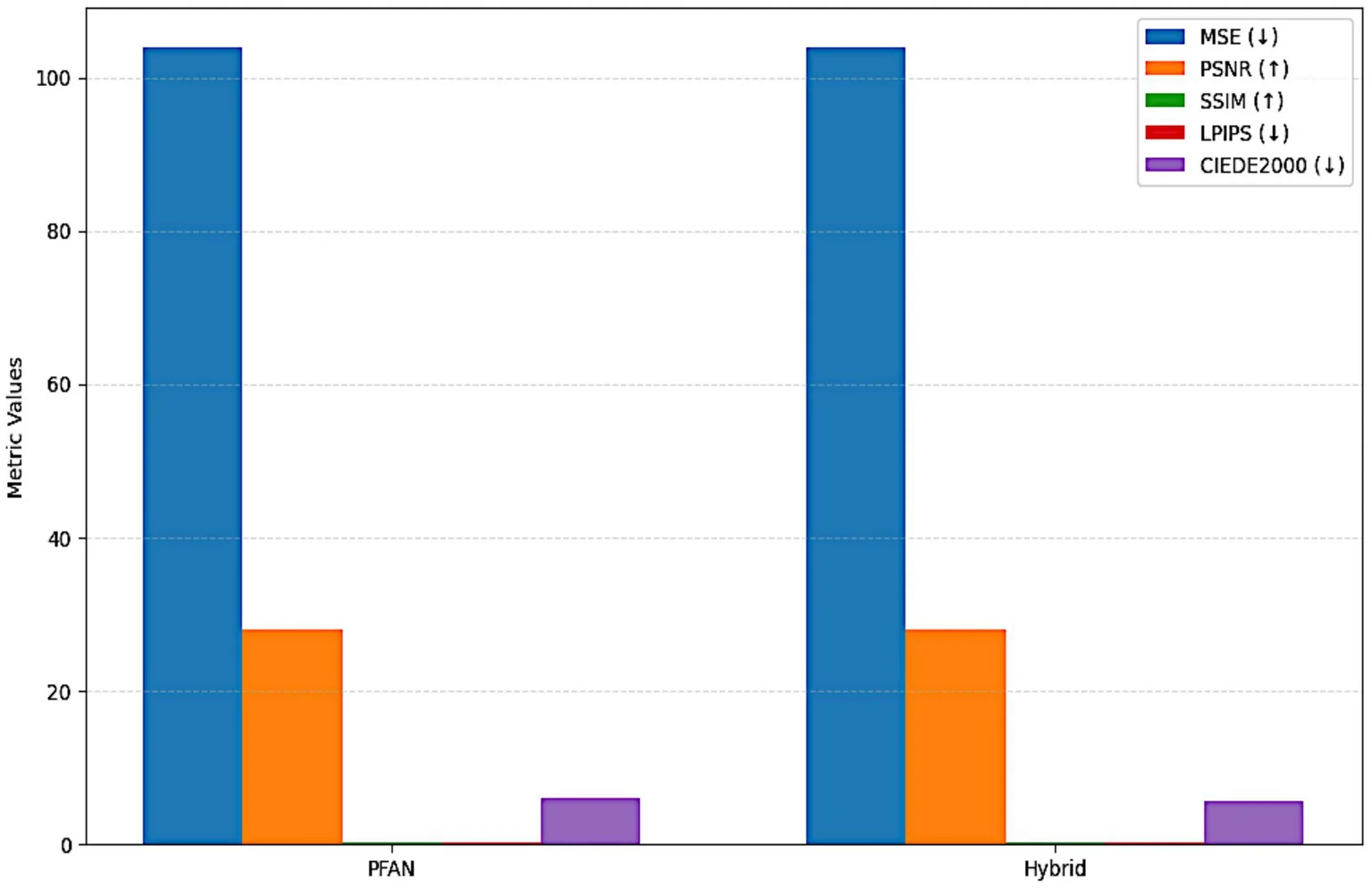
Quantitative comparison of PFAN vs. hybrid model.

The comparative study visually represents de-smoking for laparoscopy performed on representative sequences in Figure 11. For better illustration on the performance improvement, we observed from various techniques used by comparative methods that Hybrid Attention-Residual U-Net model has created a very effective de-smoking by enhancing contrast, de-noising, and clearly delineating the structures as well as reducing the unwanted artifacts while simultaneously suppressing smoke and without any structural distortion. This subjective visual observation is consistent with the objective metrics presented in Table 4.

Despite achieving enhanced restoration capabilities in the presented models while having comparatively low numbers of parameters, FPS was not assessed for evaluation in this work and was not directly compared with lightweight real-time de-smoking systems (e.g., L-SAHGNet). However, based on the small and compact model size, we assume PFAN, the hybrid attention-residual U-net to be deployable on surgical devices with limited computational capabilities. Future research will evaluate performance based on metrics like latency, throughput, memory usage, and FPS and determine real-time clinical readiness.

It is important to mention that synthetic smoke images were utilized in our quantitative assessment. Although we attempted to accurately simulate the behaviors of realistic surgical smoke through modifying the density, intensity, position, and illumination in our smoke generation technique, real surgical procedures may be more challenging to model, and the effects from unexpected conditions cannot be eliminated. Accordingly, the obtained result is limited in the present dataset, and further evaluation on clinical laparoscopic video sequences will be worthwhile to be investigated.

Moreover, the performance was also validated on the set of 660 smoke-free laparoscopic images from Cholec80 on which the synthetic smoke images were generated, which can still allow controlled and quantitative evaluation but might not capture the variations of surgical scenes, anatomy, imaging settings and smoke properties present in the clinical scenarios.

### 4.7 Limitation

The present study does not include an independent test set comprising real intraoperative smoke sequences, nor does it incorporate subjective evaluation by expert surgeons. Consequently, the reported quantitative and qualitative results should be interpreted as evidence of the effectiveness of the proposed approaches on synthetically generated smoke data only. Although the synthetic smoke generation procedure was designed to emulate realistic surgical smoke characteristics, additional validation on real clinical data and surgeon-based assessments will be necessary to establish the clinical utility and generalizability of the proposed methods.

## 5 Conclusion and future work

This paper presents analysis on de-smoking of laparoscopic images considering frequency and spatial domain-based models such as PFANs and Hybrid Attention-Residual U-Nets. In this case, it has been found that even though PFANs provide stability in training and reconstruction through frequency decomposition, hybrid attention-residual networks improve the structural consistencies and perceptual quality through residual learning and dual attention mechanisms. It has been found that both architectures produce similar PSNRs values, which range around 27.96 dB, but the hybrid networks perform significantly better in SSIMs with values of 0.296.

While the suggested method shows good potential on synthetic smoke images produced using the smoke data obtained from the Cholec80 database, this evaluation has been constrained due to lack of coupled smoke and smoke-free surgical images from the Cholec80 data set. The generation of smoke in an image artificially has the benefit that it allows regulated training and enables quantitative measurement and is also free from noise in the training data, but surgical smoke is dynamically dense in nature and varies in its shape and distribution throughout the visual field and intensity and color variations, and cannot wholly be recreated using simulated models. So the gap between the testing environment and a genuine clinic atmosphere could be established. Accordingly, future studies will evaluate the PFAN and Hybrid Attention-Residual U-Net models on real intraoperative laparoscopic recordings and patient data to assess their robustness, generalizability, and potential clinical utility.

Future work will therefore focus on the creation and evaluation of a dedicated real-smoke laparoscopic benchmark and on prospective studies involving blinded assessments by experienced surgeons. Such evaluations will provide valuable insight into visibility enhancement, preservation of anatomical structures, and the practical usefulness of the proposed de-smoking frameworks in actual surgical settings. Until such validation is completed, the present findings should be regarded as promising preclinical results rather than definitive evidence of clinical efficacy.

Moreover, while the proposed models have a rather small parameter footprint, FPS (Frames Per Second) and Latency were not considered in this work. The performance of real-time de-smoking models, like L-SAHG Net, would also serve as comparison framework. The models would be benchmarked for inference efficiency including various deploy-ready strategies such as quantization, pruning, mixed precision, and GPU execution will be conducted in subsequent research to promote a clinical trial.

In addition, one avenue for future research is to continue research in future about embedding frequency-domain and spatial-domain representations in a unified de-smoking system. Specifically, cross-domain feature fusion module could be developed to facilitate the exchanging of information between frequency-aware and spatial attention branches, thereby achieving enhanced smoke suppression while retaining the anatomical detail. Moreover, transformer-CNN co-attention can be incorporated to take advantage of global contexts and local features simultaneously. Another potential expansion of this framework could be to address real-time video de-smoking through temporal modeling of continuity, making it even more useful intraoperatively. Lastly, there is another limitation of our work that the existing dataset, Cholec80, mainly comes from the laparoscopic cholecystectomy cases, which do not reflect sufficient diversity in operative conditions, anatomical structures, visual conditions, and smoke properties compared to real-world surgeries. Therefore, to increase the generalizability, studies with diverse surgical cases will be also needed with the development of suitable domain adaption methods.

## Data Availability

The original contributions presented in the study are included in the article/supplementary material, further inquiries can be directed to the corresponding author.
